# Validation of the solution structure of dimerization domain of PRC1

**DOI:** 10.1371/journal.pone.0270572

**Published:** 2022-08-05

**Authors:** Fei Tan, Jin Xu

**Affiliations:** Peking University, Beijing, China; George Washington University, UNITED STATES

## Abstract

Cell-cycle dependent proteins are indispensible for the accurate division of cells, a group of proteins called Microtubule-associated proteins (MAPs) are important to cell division as it bind microtubules and participate with other co-factors to form the spindle midbody, which works as the workhorse of cell-division. PRC1 is a distinguishing member of MAPs, as it is a human MAP and works as the key in mediating daughter cell segregation in ana-phase and telo-phase. The physiological significance of PRC1 calls for a high resolution three-dimensional structure. The crystal structure of PRC1 was published but has low resolution (>3 Å) and incomplete sidechains, placing hurdles to understanding the structure-function relationships of PRC1, therefore, we determined the high-resolution solution structure of PRC1’s dimerization domain using NMR spectroscopy. Significant differences between the crystal structure and the solution structure can be observed, the main differences center around the N terminus and the end of the *alpha*-Helix H2. Furthermore, detailed structure analyses revealed that the hydrophobic core packing of the solution and crystal structures are also different. To validate the solution structure, we used Hydrogen-deuterium exchange experiments that address the structural discrepancies between the crystal and solution structure; we also generated mutants that are key to the differences in the crystal and solution structures, measuring its structural or thermal stability by NMR spectroscopy and Fluorescence Thermal Shift Assays. These results suggest that N terminal residues are key to the integrity of the whole protein, and the solution structure of the dimerization domain better reflects the conformation PRC1 adopted in solution conditions.

## Introduction

Cytokinesis is the last step in mitotic cell division, the whole process is coordinated by a variety of protein factors. In cytokinesis, a cleavage furrow is formed to completely divide the cell’s cytoplasm into two independent daughter cells. The microtubule arrangement in the central region of the cleavage furrow is formed by the joint action of microtubule Association proteins (MAPs) and motor proteins. Motor proteins transport MAPs to the central region of cells. MAPs bundle antiparallel microtubules through its self-polymerization ability into a relatively dense structure, and also interact with other cytokines recruited here to form the midbody, which is a complex array of microtubules and cell-factors that regulate the process of cytoplasm segregation. The loss of key MAPs will lead to the failure of the whole process, resulting in unchecked proliferation of cells, and might even lead to malignant tumors [1–3].

Protein Regulator of Cytokinesis 1 (PRC1) is a representative of MAPs that participate in the formation of spindle midbody [4, 5]. The two major functions of PRC1 are microtubule binding and self-multimerization [6, 7]. PRC1 works synergically with motor proteins (Kif4 or MKLP1) to bind microtubules and cluster them through its multimerization property [8–10]. The loss of PRC1 will lead to failure of the cell-segregating process, resulting in unchecked proliferation of cells, and might even lead to malignant tumors [1–3, 8, 11, 12].

The full-length PRC1 protein has 620 residues and is roughly divided into N-terminal polymerization domain (aa: 1–189), microtubule binding domain (aa: 190–486) and the C-terminal region (aa: 487–620). The C-terminal is structureless and extremely susceptible to degradation. Therefore, the C-terminal domain were often ignored when investigating the properties of PRC1, resulting in PRC1 1–486 (aa: 1–486) which has the same function and sub-cellular localization as the full-length protein [13].

At the junction of the central domain and the C-terminal domain, residues T470 and T481 are the sites where PRC1 is phosphorylated by CDK [10]. Zhu’s group Used colicin E3 as the basic structure model and homologously constructed a three-dimensional structure model of PRC1 in SWISS-MODEL. This model predicted that PRC1 has a G-shaped tertiary structure, and the N-terminal multimerization domain is composed of three antiparallel *alpha*-helices, This helical structure is designed to promote the multimerization of the protein. The microtubule binding domain in the central region of PRC1 consists of two antiparallel domains of *alpha*-helical composition. The C-terminal structure of the protein folds back to the N-terminal and is close to the N-terminal multimerization domain, so that the two CDK phosphorylation sites are close to the N-terminal multimerization domain. The authors believed that the phosphorylation and dephosphorylation of PRC1 protein not only affect the binding of PRC1 to motor protein Kif4, but also relate to its own multimerization [7].

The crystal structure of PRC1 1–486 (PDB ID 4L6Y) showed that it is divided into the dimerization domain (aa: 1–66), the rod domain (aa: 67–350) and the spectrin domain (aa: 351–486). These three domains are composed of 9 *alpha*-helices, extensive hydrophobic interactions between the helices maintained the stability of the whole structure. The length of the monomer protein is 22 nm. When the protein dimerizes, the two monomers interact with each other by the N-terminal dimerization domain. The dimer protein can reach 32 nm in length which is consistent with the microtubule spacing of 35 nm [13, 14].

The rod domain comprises a 70 Å long three-helical strand connected by loops. All residues located in this three-helical bundle participate in the formation of a hydrophobic core interface, this domain is also responsible for PRC1 microtubule binding through a stretch of conservative and positively charged residues. The dimerization domain exhibits a helix-turn-helix motif, forming a U-shaped hairpin with two helices H1, H2 and one loop. Upon dimerization, two U-shaped hairpins are inserted into their respective hydrophobic centers to form a four-helix bundle. Subramanian’s group Reconstructed the three-dimensional structure of PRC1 using Cryogenic Electron microscopy, but the electron density of dimerization domain is not found in the reconstructed structures, they believed that this indicate that the dimerization domain is likely to have dynamic flexibility and might show multiple conformations in different conditions [13, 15].

PRC1 can form transverse bridges between adjacent microtubules to aggregate microtubules into bundles through its self-multimerization property. Many research groups pointed out that cell-cycle dependent phosphorylation and dephosphorylation of PRC1 can regulate its polymerization state. Zhu’s group found that the molecular weight of PRC1 protein was about 300KDa in non-phosphorylated state, indicating that PRC1 may form tetramers when dephosphorylated, but they also showed that PRC1 exist as only a monomer in the phosphorylated state [7]. Mollinari observed excessive oligomerization of PRC1 protein in non-phosphorylation state, resulting in excessive aggregation of cell spindles and affecting cytokinesis [16]. Fu’s group observed that dephosphorylated PRC1 is a mixture of tetramers and dimers [17]. Subramanian’s group determined the multimerization state of PRC1 in vivo using fluorescence strength analysis, and found that the multimerization state of PRC1 is not affected by phosphorylation [13]. The phosphorylation state of PRC1 mainly affect its multimerization through interaction of the phosphoryaltion site and the N terminus multimerization domain.The conflicting reports about the multimerization state of PRC1 highlight the need for more research on the multimerization state of PRC1.

A more detailed and precise determination of protein solution structure is crucial for better understanding the protein relationship-function under solution conditions. Because the crystal structure is incomplete in sidechains, has low resolution (3.1 Å) and showing higher than average B-factors in part of the protein (S11 Fig), these reasons propagate us to determine the solution structure of PRC1. We have determined the solution structure of PRC1 N terminus dimerization domain using Nuclear Magnetic Resonance (NMR) Spectroscopy. PRC1 dimerization domain (also known as PRC1-DD, aa:1–65), is a stable homodimer with a molecular mass of 16kDa (S1–S3 Figs), it is an independent domain with little or no interaction with the distal part of PRC1. We have reported the solution structure of PRC1-DD, which is generally similar with the crystal structure in shape, but very different in folding and hydrophobic core composition. When analyzing the differences between the solution and crystal structure, we found that one of the most distinctive structural traits of the solution structure is its N terminus, in which the first residue M1 is folded into the hydrophobic core, while being outside of the hydrophobic core in the crystal structure. The N terminus of most proteins are exposed or located outside of the hydrophobic core, but for some proteins such as SARS-CoV Main protease [18, 19], Xylanase (BSX) from *Bacillus sp*. [20, 21], Flock house virus (FHV) coat protein [22], etc. [23, 24], the N terminus play crucial roles in the function or the structural integrity of the protein. Seven residues in the N terminus of SARS-CoV Main protease is indispensable for the protein’s enzymatic activity, disruption in its N terminal residue composition will lead to structurally deformed protein or reduced viral coat cleavage. The N terminal residues of BSX is pivotal to the protein’s resistance to degradation by proteinase and surface active agents, deletion of its N terminus renders the protein susceptible to degradation. The N terminus of FHV coat proteins contain recognition sequences for the recognition and packaging of viral RNA, the deletion of its N terminus residues produces deformed viruses. For PRC1-DD, we have measured the change in the structure of the protein when adding or deleting residues from PRC1 N terminus using mutational studies [25–30], and tracked changes in its thermodynamic properties using Fluorescent Thermal Shift Assay (FTSA). We found that deletion of the first residue M1, brought substantial changes to overall structure of the protein, indicative of a perturbed hydrophobic core; addition of extra residue in the N terminus of PRC1 also caused huge structural variations to the whole protein. The thermal stability of N terminal deleted or extended mutants were also significantly reduced compared to wild-type proteins. Therefore, we conclude that the N terminus of PRC1 is crucial for the structure and folding of the whole protein, and deletion or addition to the N terminus will affect the whole protein either thermodynamically or structurally [31].

## Materials and methods

### Protein-template construction

The nucleic acid sequences of PRC1 1–486 and PRC1-DD were amplified from human-PRC1-isoform-1 (Pubmed accession: NP_003972) and inserted into the bacterial expression vector pET-21a containing a C-terminal His-tag. The detailed protocol is as follows:

1. DNA fragments encoding PRC1 1–486 and PRC1-DD were amplified by polymerase chain reaction (PCR) with primer sequences, as shown in S4 Table.
2. The PCR products and the circular pET-21a vector were digested by restriction enzymes NDE I and Xho I (NEB), respectively, to obtain target-gene insertion fragments with sticky ends and the corresponding linear plasmids.
3. The insertion fragments of the target gene were incorporated into the linear plasmid vector by T4 ligase and the product was transferred to E. coli competent cell TOP10. The positive strain was screened by ampicillin resistance, and PCR confirmation and DNA fragment sequencing were carried out.
4. The recombinant plasmids with correct sequencing were obtained using a plasmid extraction kit (Invitrogen).

### Mutant-plasmid generation

1. Mutant proteins of PRC1 1–486 and PRC1-DD were generated using a QuickChange Mutagenesis kit (Agilent) with the following reaction system: 200 ng of forward primer (provided in S4 Table), 1 *μ*L of reverse primer (provided in S4 Table), 1 *μ*L of 5 *reaction buffer, 10 *μ*L of 2.5 mm dNTPs, 4 *μ*L of Pfu DNA polymerase and 1 *μ*L of Nuclease-free water, for a total of 50 *μ*L.
2. We set the PCR reaction conditions according to the instructions of the Pfu DNA polymerase for 30 cycles.
3. We added DPN 1 restriction endonuclease to the reaction system and incubated it at 37 ° for 1 hour.
4. We tested the products by electrophoresis and selected the DNA band with the correct length and used a DNA gel Recovery Kit (Zymo Research) to obtain the target plasmid; then, we used 100 ng of the product for conversion into E. coli competent cell TOP10. Antibiotic agar plates for positive-strain selection were used.
5. Single colonies were picked and sent for sequencing. The correct plasmids were used for mutant protein expression.

### Protein expression

Expression of wild-type PRC1-DD, PRC1 1–486 and mutant proteins were carried out in E. *coli* BL21 (DE3) or *Rosseta* (DE3) cells. Proteins were expressed for 6–20 hours at 18 °C–35 °C with 500 mM IPTG and then purified in accordance with the following protocol:

1. The recombinant plasmids encoding the PRC1 target proteins were transformed into E. *coli* competent cells (both BL21 (DE3) and *Rosseta*); the plates were coated with agar containing ampicillin and incubated at 37 °C.
2. Single colonies were selected and inoculated into 40 ml of Luria Bertani (LB) medium containing ampicillin, then incubated overnight at 35 °C.
3. We transferred the bacterial solution into 1 L of fresh LB medium containing ampicillin and cultured it at 35 °C to OD 600; then, we added 0.05 g of IPTG to induce the expression of the target protein at 18 °C for 6–20 h.
4. After continuous culturing for 6 h, we centrifuged the bacterial solution at 7000 rpm for 15 min, poured out the supernatant and resuspended the bacteria with 30 ml of liquid buffer containing 50 mM PBS and 300 mM NaCl, pH 8.0; then, we froze it at −80 °C.

### Protein purification

1. E. *coli* cells were harvested by centrifugation, resuspended in lysis buffer (50 mM PBS, 300 mM NaCl, 5% glycerol and 25 mM imidazole, pH 8.0) supplemented with complete protease inhibitor cocktail (Roche, US) and then sonicated.
2. After separation of supernatant and pellet by centrifugation, the supernatant was loaded onto a His-Trap HP column (Tiagen); by applying a linear imidazole gradient (20–300 mM), we pooled the His-tagged proteins.
3. The proteins were then further purified by size-exclusion chromatography on a Superdex 75 or Superdex 200 column (GE Healthcare Life Sciences) equilibrated at 50 mM PBS and 150 mM NaCl, pH 7.0. Fractions corresponding to the target proteins, as confirmed by SDS-PAGE, were pooled, concentrated and stored at -80 °C. The purity of all protein preparations was greater than 95% based on polyacrylamide gel electrophoresis in the presence of DTT.

### Labeled-protein preparation

We used E. *coli* BL21 (DE3) or *Rosseta* cells for labeled-protein expression. To prepare ^15^*N*-labeled or ^15^*N*/^13^*C*-labeled protein for NMR studies, we grew cells in M9 minimal medium with ampicillin (100 mg/L) and ^15^*NH*_4_*Cl* in the absence or presence of ^13^*C*-glucose for the generation of ^15^*N*-labeled or ^15^*N*–^13^*C*-double-labeled samples [32].

After overnight incubation at 37 °C in LB growth medium, the cells were added in a 1:20 ratio by volume to 1 liter of LB medium. After cells reached an absorbance of 0.8–1.0 at 600 nm (around 4 hours at 37 °C), they were added to 500 ml of M9 minimal medium with ^15^*NH*_4_*Cl* and D-glucose-1,2,3,4,5,6,6-d7 as the sole nitrogen or carbon sources. All media contained 100 mg/L ampicillin. After the cells had grown at 37 °C to an absorbance of 0.8 at 600 nm, the temperature was decreased to 18 °C and IPTG was added at a concentration of 100 mg/l. After 6–20 hours of protein expression, cells were harvested by centrifugation. The PRC1 protein was then extracted from cells and purified.

### High-performance chromatography

A high-pressure liquid chromatography system was equipped with a UV detector (Hitachi, Japan). The HPLC columns were Superdex75 5/150 and Superdex200 5/150 (GE Healthcare Life Sciences).

All solvents (methanol, acetonitrile, hexane, propanol and ethanol) were HPLC grade, purified and degassed before use.

The protein sample (<10mg) was dissolved in effluent and injected into the column with a flow rate of 0.3–1 ml, as recommended by the column manual, and before injection, all samples were centrifuged at 10,000 g for 10 minutes. All figures and molecular mass measurements were generated directly by the device’s default software.

### Chemical cross-linking

Cross-linking studies were carried out using PEG5 as cross-linker, which cross-links every possible NH ester within 23 Å. Prior to cross-linking, the purified proteins were kept in buffer containing 50 mM PBS and 50 mM NaCl, pH 7.0. Sample concentration was adjusted to 20 nM and 50 nM, then cross-linking reactions were carried out at 25 °C with 0.1 M-1 M PEG5 reagent (ThermoFisher Scientific). Aliquots were removed after 30 min of incubation and reaction-quenched by the addition of 1M Tris-HCl, to a final concentration of 50 mM. The reaction results were tested by SDS-PAGE [33].

### NMR samples preparation

NMR samples were prepared in 50 mM PBS, 150 mM NaCl, 5 mM DTT, 0.01% DSS and 5% *D*_2_*O*, pH 7.4, and were between 0.5 mM and 1.0 mM in concentration. Isotopic heterodimers were prepared by mixing equal amounts of unlabeled and ^15^*N*–^13^*C*-double-labeled PRC1-DD (16 mg each) in 2 ml of 8 M urea for several minutes and then dialyzing by ultracentrifugation (Amicon Ultra Centrifugal Filters; 10,000 molecular weight cutoff). The samples were then concentrated to 1 mM. All steps were performed at 4 °C.

### Fluorescence-Based Thermal Shift Assay (FTSA)

Thermal shift assays were performed using a Real-Time PCR Detection System (StepOne Real-Time PCR System; Applied biotechnology, CA, USA) with a temperature increment of 0.2 °C and a temperature range of 25–95 °C. A total of 25 *μ*L of mixtures containing 2.5 *μ*L of protein dye (Protein Thermal Shift Starter Kit; Life Technologies, Carlsbad, CA, USA; diluted from 5000x concentrate stock), 10*μ*L of reaction buffer (Protein Thermal Shift Starter Kit) and 12.5 *μ*L of protein (at a concentration of 0.5 mM), was mixed on ice in a 96-well plate. The mid-denaturation temperatures (Tm) that measure protein folding and unfolding transitions were estimated using the device with the following equation [34, 35]:

$$\begin{array}{c} I=(A+\frac{B-A}{1+e^{(T_{m}-T)/C}}) \end{array}$$ (1)

where *I* is the fluorescence intensity at temperature *T*, *A* and *B* are the pre-transitional and post-transitional fluorescence intensities, respectively, and *C* is a slope factor.

### Static light scattering and SEC-MALS

The statistical analyses were carried out using the paired two-sample Student’s t-test for means in Excel, comparing each mutant with the wild type; the p-value thresholds were set at 0.05 for significant and at 0.01 for very significant.

### Hydrogen deuterium exchange experiments

Lyophilized 15^*N*^-labeled PRC1-DD monomer samples were dissolved in the deuterated buffer containing 50 mM PBS (pD 7.0) with 1 mM DTT and 1 mM EDTA, and were immediately used for NMR analysis on a Bruker Avance 500 MHz spectrometer (with Cryoprobe). A series of two-dimensional ^1^*H*-^15^*N* HSQC spectra were collected to monitor the NH signal intensity decrease as a function of time at room temperature. Hydrogen exchange rate constants (*k*_*ex*_) were determined according to reference [36] by fitting the experimental data to a single exponential function using Matlab:

$$\begin{array}{c} I=I_{0}exp\left(-k_{ex}t\right)+C \end{array}$$ (2)

where C stands for the baseline noise caused by residual water in the sample [37], *I*_0_ is the initial intensity factor.

Then the apparent free energy of hydrogen exchange Δ*G*_*ex*_, was determined according to:

$$\begin{array}{c} \Delta G_{ex}\left(T\right)=-RTln\left(k_{ex}/k_{rc}\right)=-RTln\left(1/PF\right) \end{array}$$ (3)

where *PF* is the protection factor for a given backbone amide of every residue, *k*_*rc*_ is the value for the intrinsic exchange rate constant obtained using the online program SPHERE (http://www.fccc.edu/research/labs/roder/sphere/). The values of *k*_*rc*_ of consecutive residues are provided in Supporting information.

### Statistical analysis

Statistical analyses were carried out using the paired two sample for means student t-test in excel, comparing each mutant with wild-type, p value thresholds were set as 0.05 for significant, 0.01 for very significant.

## Results

We used PRC1-DD for structural determination and analysis, The dimerization domain is an independent domain, with little or no interactions with the distal part of PRC1 full-length protein. Through measurements by various methods such as chemical cross-linking, size-exclusion chromatography and static-light-scattering analysis (S1 and S2 Figs) and Small Angle X-ray diffraction (SAX) PRC1-DD was determined to be homo-dimers in solution with 65 residues per subunit and a molecular mass of around 15kDa.

### Three dimensional structure of PRC1-DD

**Fig 1:**
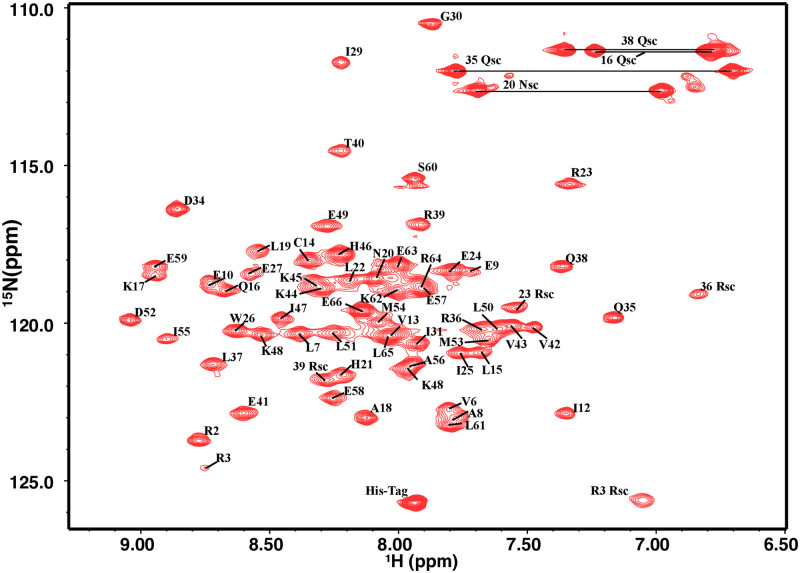
Two-dimensional ^1^*H*-^15^*N* HSQC spectrum and assignment of PRC1-DD.

After obtaining a total of 3717 distance constraints, 256 dihedral angle constraints and 120 intermolecular distance constraints, etc. using NMR spectroscopy, we determined PRC1-DD to be an homodimeric, each subunit has 65 residues forming two *alpha*-helix and a loop (Fig 1). The distance constraints used to determine the intermolecular structure and composition between subunits mainly come from Three-dimensional ^15^*N* and ^13^*C* NOESY-HSQC spectra and Three-dimensional ^1^*H*-^13^*C* filtered NOESY-HSQC spectra (S5 Fig and S3 Table). We obtained the ensemble of 20 most energetically favorable structures. Using Procheck [38] to analyze these structures, 100.0% of the residues are located in the optimal region (96%) or additional allowable region (4.0%) of the Ramachandran diagram [39], the structural accuracy is equivalent to the X-ray crystal structure with a resolution of 1.5 Å.

Homodimeric PRC1-DD contains two subunits. Each subunit is a U-shaped hairpin containing 65 residues, which is formed by *alpha*-Helix H1 (residues E3-l28) and *alpha*-Helix H2 (D34-R65) connected by one loop (I29-E33).

**Fig 2:**
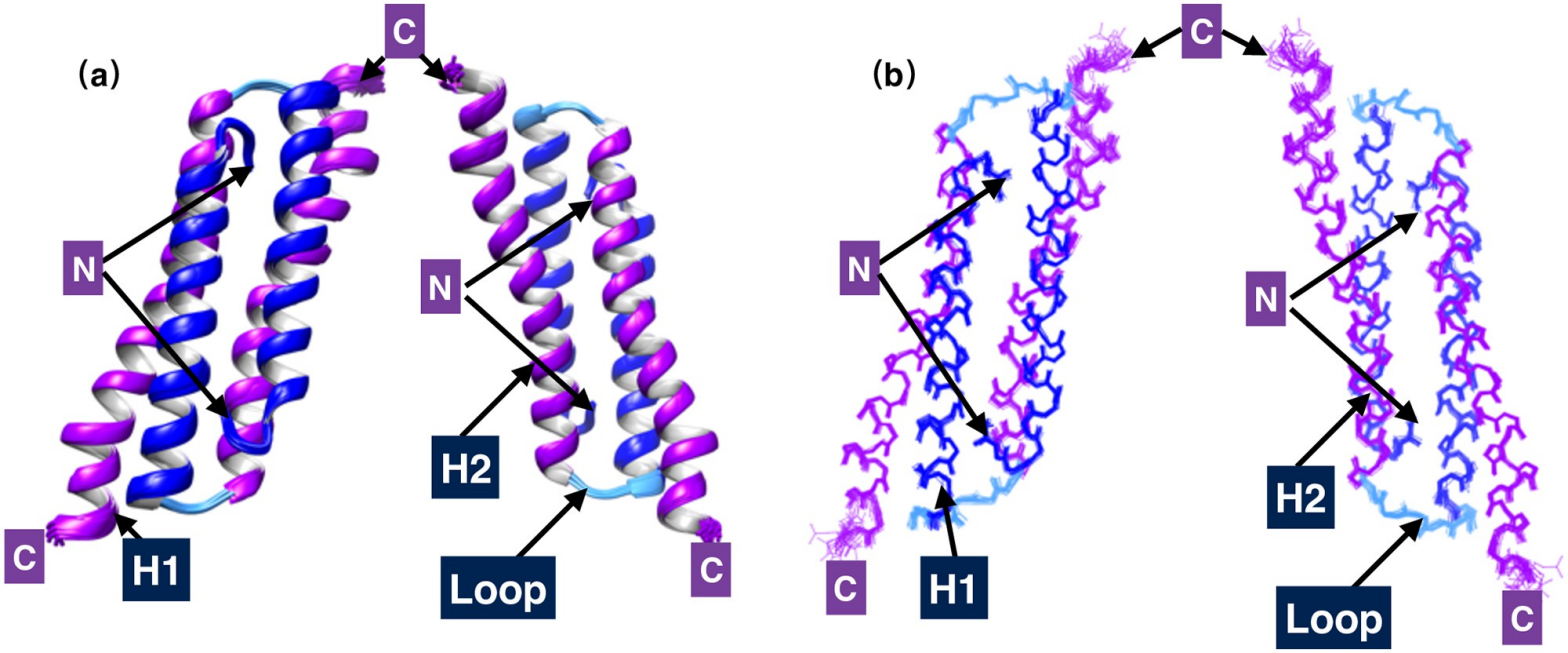
Solution structures of PRC1-DD. **(a)** Ribbon representation of the 20 lowest-energy structures. **(b)** Backbone chain trace of the 20 lowest-energy structures; helices H1 and H2 from the two monomeric units in the homodimer are colored separately in blue (H1, M1-E28), cyan (loop, I29-D32) and purple (H2, E34-L65); C and N tags signify C- and N-terminal ends, respectively. This and all other structural figures were generated using the program CHIMERA.

The H1s and H2s of the two subunits in PRC1-DD are inserted into each other’s centers in a symmetrical manner to form a four-helix bundle. A large hydrophobic core is in the center of the four-helix bundle, which is composed of hydrophobic residues such as L15, L22, v43, L50, M53 and E57. The backbone heavy atom RMSD (Root mean Square Deviation) of the 20 structure ensemble is 0.49 Å, and the RMSD of all heavy atoms is equal to 1.06 Å. Similarly, the two subunits in PRC1-DD overlap well. The RMSD values of the main chain heavy atom and all heavy atoms between the two subunits are 0.43 Å and 1.10 Å, respectively. Two different subunits in the homologous dimer can overlap well, indicating that the dimer interface is clearly defined, A ribbon representation of the structure ensemble is given in Fig 2.

### Comparison between solution structure and corresponding segment of crystal structure

**Fig 3:**
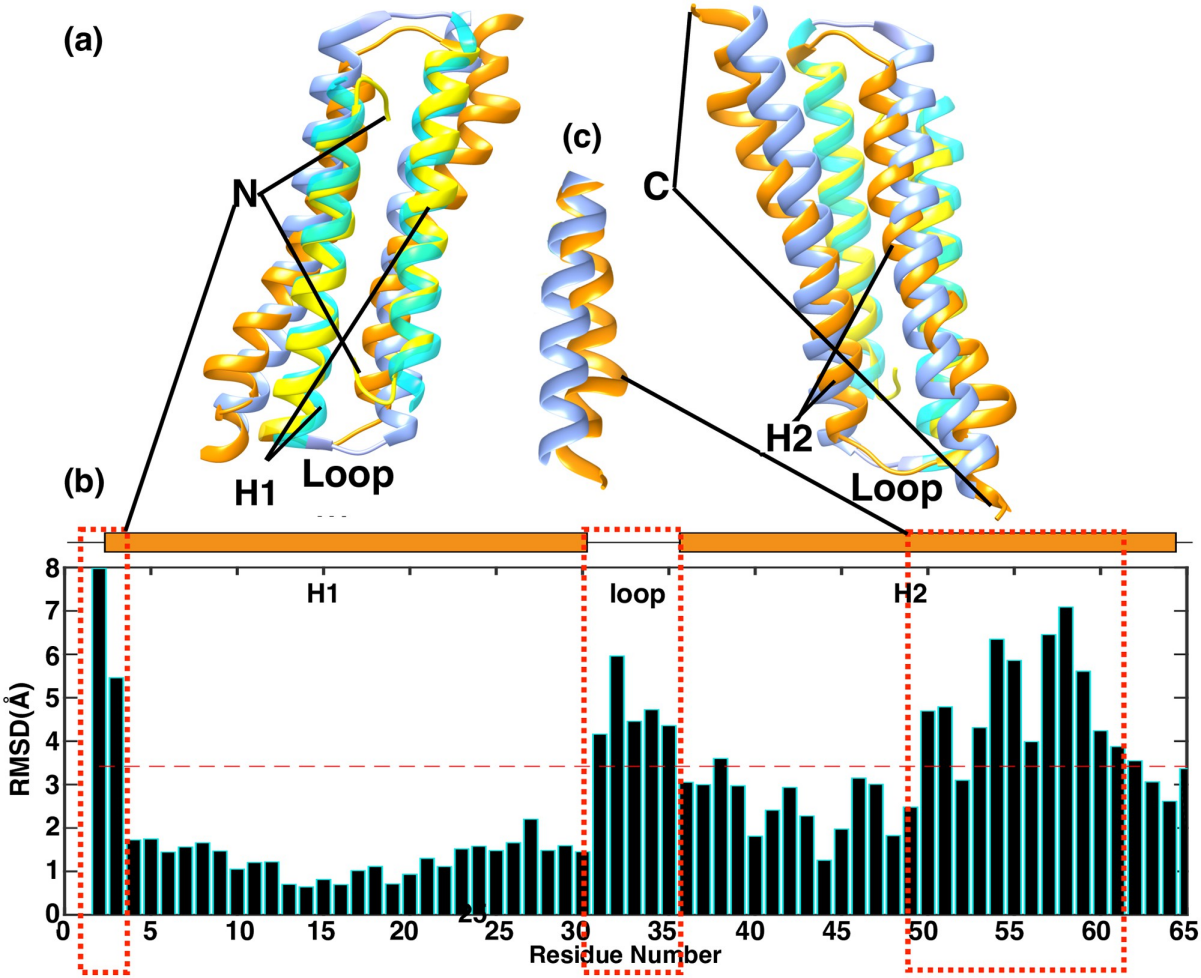
Comparison of the solution structure of PRC1-DD and the corresponding segments in crystal structure. **(a)** Superimposition of ribbon representation of the PRC1-DD lowest-energy solution structure and crystal structure; H1 and H2 of the solution structure are colored yellow and orange, respectively, while H1 and H2 of the crystal structure are colored in cyan and blue, respectively; C and N tags signify C- and N-terminal ends, respectively. **(c)** Enlargement of the dotted box in **(a)**. Figures were plotted using MATLAB, while the alignment was performed using CHIMERA.

The general shape of the solution structure of PRC1-DD and the corresponding segment of PRC1 crystal structure are similar, but close inspection showed significant differences (Fig 3(a)). In terms of secondary structure, in the crystal structure, *alpha*-helix H1 starts from R3, *alpha*-Helix H2 starts at E33, while according to the solution structure, H1 starts from E5, H2 starts from D34. RMSD of the solution structure compared to the crystal structure is 3.48 Å, which shows that there is a large difference in the overall structure between the two. As shown in Fig 3(b), The average RMSD of residues R2-E28 (H1) is 1.67 Å, while that of residues K34-R62 is 3.67 Å, indicating that the solution structure and crystal structure at the end of H2 are quite different. We found that the structural regions with the most obvious structural differences include the N-terminal, loop region, and end regions of H2. In the solution structure, the N-terminal residue M1 is inserted into the hydrophobic core of PRC1-DD, while in the crystal structure, M1 is located outside the hydrophobic core, the RMSD between them is as high as 7.51 Å. The loop region also has a high RMSD of 4.01 Å. The conformation of the loop region in the solution structure is different from that of the crystal structure, which may lead to different orientations of H2.

In addition, the solution structure and crystal structure of PRC1-DD have different dimerization interfaces. When aligning the crystal structure and solution structure of one monomer, the average RMSD value of this monomer is 2.61 Å and the RMSD value of the other monomer is up to 5.72 Å (S4(b) Fig). When the dimeric interfaces of the two monomers are the same, the average RMSD value of the two monomers should be the same compared with a single subunit. These comparisons show that the crystal structure and solution structure of PRC1-DD have different local structures in each monomer, and their dimerization interfaces are also distinctive.

**Fig 4:**
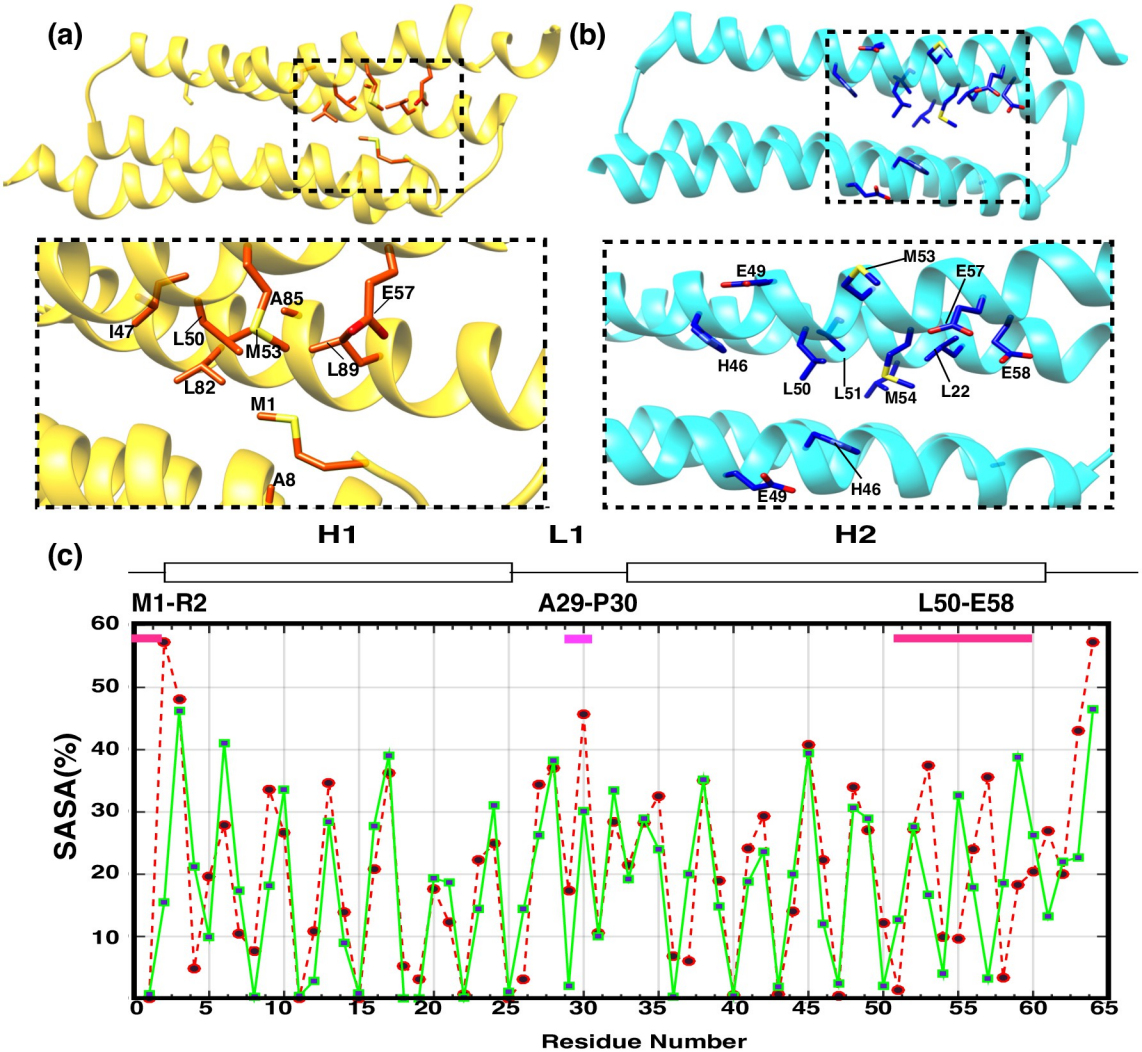
Differences between solution and crystal structures. **(a)–(b)** The hydrophobic-core composition of **(a)** solution and **(b)** crystal structures with residues having significant differences being tagged. **(c)** Relative solvent-accessible surface area (SASA) as a function of residue number (x-axis); each residue SASA value is represented as a black dot; green continuous line belongs to the SASA values of the crystal structure, while dotted red line represents solution-structure SASA values.

The composition of protein hydrophobic cores are also significantly different among the solution and crystal structures. The residues with great differences in hydrophobicity and side-chain conformation in the solution structure and the crystal structure are mainly centered around (Fig 4(a))

- The N-terminal residues M1 and R2 of PRC1-DD, M1 folds into the hydrophobic core of the solution structure, while on the contrary, the first residue M1 in the crystal structure is located outside of hydrophobic core.
- Some residues on *alpha*-helix H2, such as E49, L50, L51, M53, M54, E57 and E58, have different conformations in the solution and crystal structures. Residues M1, L50, M53 and E57 are located inside the hydrophobic core in the solution structure, while residues L51, M54 and E58 are located outside the hydrophobic core. In the crystal structure, the hydrophobic core is composed of residues such as L51, M54 and E58, and M1, L50, M53 and E57, locating outside the hydrophobic core.

The solvent accessibility values of residues such as M1, R36, L37, L50, L51, E57, E58, I55 and A56 vary greatly among the solution and crystal structure. Among them, residues M1, R36, M53, L50 and E57 are in the hydrophobic core in the solution structure, and their solvent accessibility values are smaller according to the solution structure, while these residues are outside the hydrophobic core in the crystal structure, and their solvent accessibility values are larger compared to that of the solution structure. On the contrary, L37, L51, M54, I55 and E58 are located in the hydrophobic core in the crystal structure, and their solvent accessibility values are smaller, while these residues are located outside the hydrophobic core in the solution structure, and their solvent accessibility values are larger (Fig 4(b)). This indicate that the residues involved in the formation of hydrophobic core of the solution structure and crystal structure of PRC-DD are entirely different.

### Validation of the solution structure of PRC1-DD by measuring hydrogen-deuterium exchange kinetics

**Fig 5:**
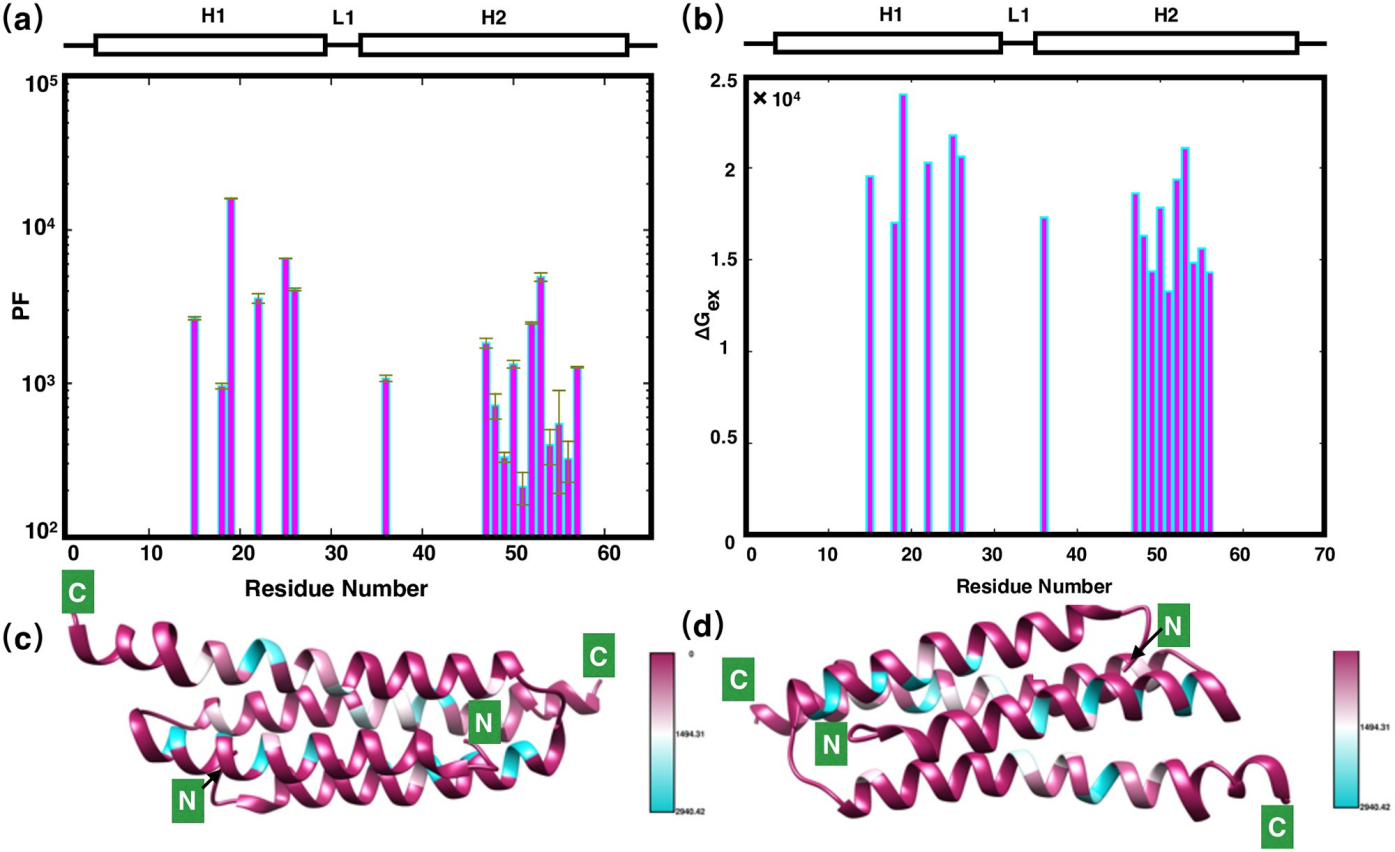
H/D exchange of PRC1-DD. **(a)** Bar graph showing the protection factor of each amide proton in PRC1-DD, schematic representations of PRC1-DD structure were also provided on top of the figure. **(b)** Bar graph showing the unfolding free energy (Δ*Gex*) of each residue. **(c)** Experimentally determined free energy of unfolding (Δ*Gex*) mapped to PRC1-DD solution structure. The Δ*G*_*ex*_ ranges are: fast exchangers (purple) Δ*Gex* ≥ 30000; medium exchangers (white) Δ*G*_*ex*_ = 15000–30000; slow exchangers (cyan) Δ*G*_*ex*_ ≤ 15000. Figures were drawn using CHIMERA.

Hydrogen deuterium exchange (HDX) experiment measures the exchange rate between backbone amides of residues and deuterated solvent. In HDX experiments, hydrogen bonded or buried residues exchange with solvent significantly slower than non-hydrogen bonded or exposed residues [40]. HDX exchange kinetics of residues can reflect the hydrogen-bonding pattern of a specific local region, and were also a good assessment of residue-specific hydrophobicity. Here, Residue-specific amide HDX exchange rates, and its corresponding protection factors were calculated according to [36], all these values are shown in Table 1 and mapped to structure in Fig 5(c).

**Table 1:** Hydrogen-deuterium exchange data of PRC1-DD backbone amides.

Residue	*k*_*ex*_(*min*^−1^)	PF	Δ*G*_*ex*_(J *mol*^−1^)
15L	1.82 × 10^−3^	2.66 × 10^3^	1.95 × 10^4^
18A	1.88 × 10^−3^	9.59 × 10^2^	1.70 × 10^4^
19L	8.09 × 10^−4^	1.61 × 10^4^	2.40 × 10^4^
22L	1.41 × 10^−3^	3.59 × 10^3^	2.03 × 10^4^
25I	2.40 × 10^−4^	6.54 × 10^3^	2.18 × 10^4^
26W	4.00 × 10^−4^	4.10 × 10^3^	2.06 × 10^4^
36R	2.77 × 10^−3^	1.08 × 10^3^	1.73 × 10^4^
47I	2.00 × 10^−3^	1.83 × 10^3^	1.86 × 10^4^
48K	3.88 × 10^−3^	7.19 × 10^2^	1.63 × 10^4^
49E	3.88 × 10^−3^	3.30 × 10^2^	1.44 × 10^4^
50L	8.31 × 10^−4^	1.34 × 10^3^	1.78 × 10^4^
51L	1.00 × 10^−2^	2.12 × 10^2^	1.33 × 10^4^
52D	1.79 × 10^−3^	2.48 × 10^3^	1.94 × 10^4^
53M	1.74 × 10^−3^	4.95 × 10^3^	2.11 × 10^4^
54M	4.13 × 10^−3^	3.97 × 10^2^	1.48 × 10^4^
55I	6.58 × 10^−3^	5.45 × 10^3^	1.56 × 10^4^
56A	7.40 × 10^−3^	3.22 × 10^3^	1.43 × 10^4^

HDX experimental results can be a further proof for the differences in solution and crystal structures. For a protein like PRC1-DD, in which almost all helix-composing residues possess the same hydrogen bonding pattern, HDX exchange rates of backbone amides of such a protein should be proportional to each residue’s solvent accessible surface area (SASA). When examining the relative SASA values for each residue (Fig 4(c) and S2 Table) in the solution and crystal structures, we found that the SASA values of residues A18, L19, L50, L51, M53, M54 and I55 differ significantly, and the H/D exchange rates of these residues also differed accordingly. Further analysis revealed that the H/D exchange rates on these residues might be key to confirm the validity of solution structure. For instance, for residue pair A18 and L19, in the solution structure, L19 is suggested by its SASA as the more protected residue compared to A18, however, the SASA of crystal structure indicate that A18 is more buried than L19. The solution structure is thus confirmed by HDX data in which L19 possess lower HDX rate and thus should be more buried. Furthermore, for residue pair L50 and L51, L50 is the more solvent-exposed residue according to the solution structure while in the crystal structure, it is L51 that is more buried, accordingly, the solution structure is once again substantiated by HDX data in which the exchange rate of L50 is almost an order of magnitude smaller than L51. Likewise, for residue pair M53 and M54, the crystal structure indicates that M54 is the more solvent-exposed residue while it is M53 that is more buried according to the solution structure. HDX rates confirmed that M53 is the more solvent accessible residue with its ten times lower HDX rate constant compared to that of M54. In sum, the HDX kinetics confirmed the validity of solution structure.

### Validation of solution structure by mutating key residues

Since all the differences between the solution structure and the crystal structure were possibly the result of the special conformation adopted by the N-terminal residues, we employed mutational studies and fluorescence thermal shift assays to reveal the exact conformation of the PRC1 N terminus under solution conditions. The principles for choosing mutational sites are as follows:

- Residues that in the solution structure are crucial for protein hydrophobic core formation while at the same time, outside of crystal structure hydrophobic core.
- Residues that are trivial to the integrity of solution structure hydrophobic core but instead participate in core formation according to the crystal structure.

#### N terminus residues

**Fig 6:**
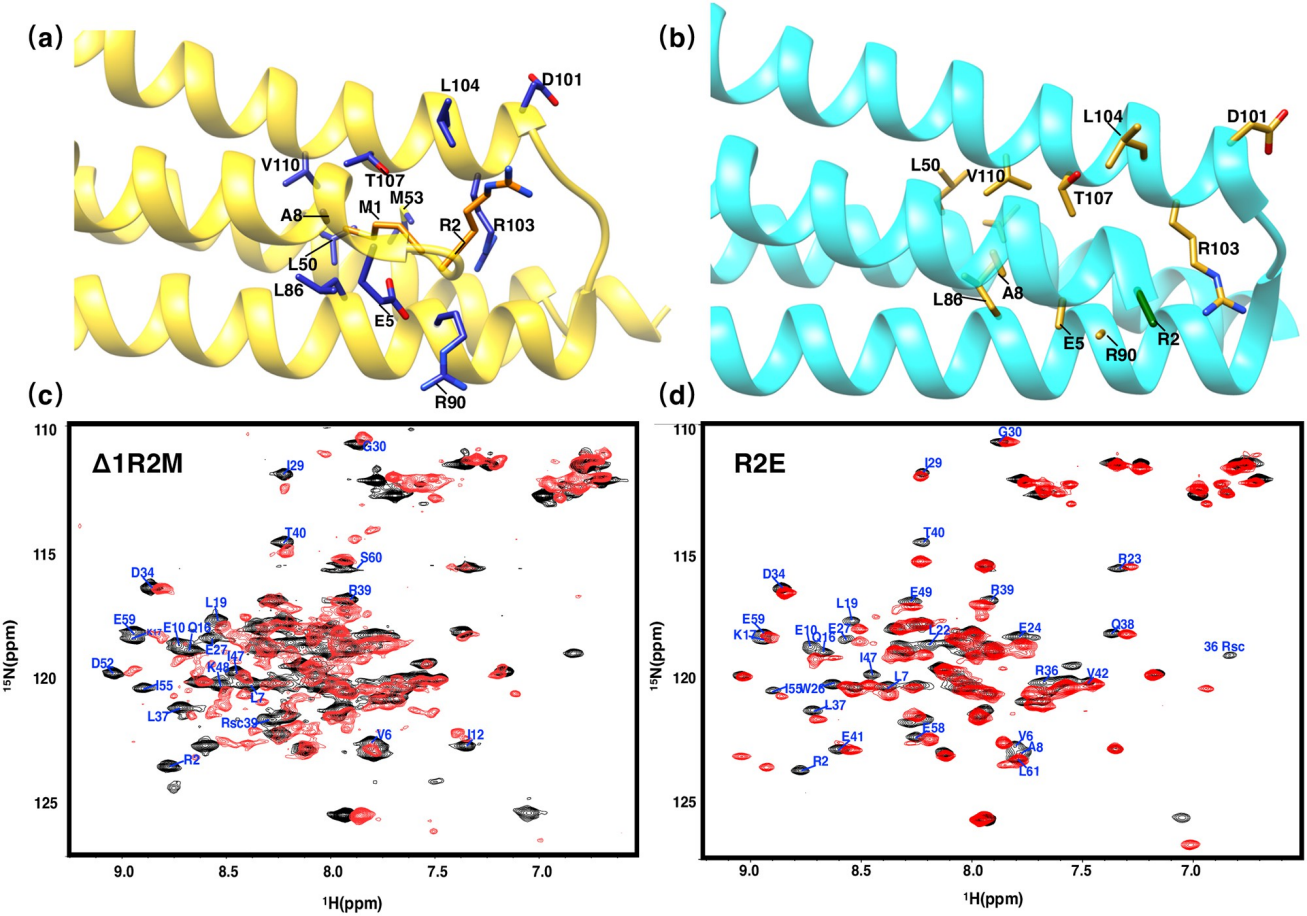
Two-dimensional ^1^*H*-^15^*N* HSQC spectra of Δ1R2M and R2E. Ribbon representation of the **(a)** solution structure (gold) and **(b)** crystal structure (cyan) with the side chains M1 and R2 in orange and green, while the side chains of the residues interacting with M1 or R2 are shown in blue and mud yellow for solution and crystal structures, respectively. **(c)** Two-dimensional ^1^*H*-^15^*N* HSQC spectra of Δ1R2M. **(d)** Two-dimensional ^1^*H*-^15^*N* HSQC spectra of R2E, residues experiencing great chemical-shift changes (Δ comp ≥ 0.3 ppm) are labeled.

The solution structure and crystal structure of PRC1-DD have significant differences in the structural conformations of N-terminal residues M1 and R2. Residue M1 in the solution structure is inserted into the hydrophobic core and has hydrophobic interaction with many hydrophobic core residues. In the solution structure, the sidechain of M1 interact with the *alpha*-proton of L50, the *beta*-proton of M53, the *alpha*-proton of M54 and the *beta*-proton of R39 in the other subunit. This unique structural feature mediates the packaging of hydrophobic core, while residue M1 is completely outside of the hydrophobic core in crystal structure. In order to assess the importance of N terminal residues, we generated mutants 1–2ΔR3M and 1ΔR2M which deleted residue 1–2 and changed residue R3 to M and deleted residue M1 and changed R2 to methionine, respectively, If M1 is not located inside the protein hydrophobic core, but is otherwise left outside of the protein hydrophobic core, changing it may have little impact on the overall folding or the structure of the protein. We did not observe soluble expression for 1–2ΔR3M, suggesting that the N terminal residues is in fact important for protein folding while according to the two-dimensional ^1^*H*-^15^*N* HSQC of 1ΔR2M, the chemical shifts of the cross peaks of most residues (70%) changed greatly (Δ comp ≥ 0.3 ppm), based on this, we can speculate that the hydrophobic core of the whole protein is disturbed (Fig 6). Residues that are close to or may interact with M1 in the solution structure, such as R3, A8, L19, T40, R36, L22, L37, R39, V47, M53 and M54, all experienced significant chemical shift disturbances according to the two-dimensional ^1^*H*-^15^*N* HSQC spectra. Therefore, we can conclude that the overall structure of PRC1-DD has undergone great structural changes due to the removal of M1 (S6 Fig).

Generally speaking, mutating charged residues may have great impacts on the overall structure of the protein, to overrule the possibility that the structural changes caused by 1ΔR2M are due to the mutation of R2. We mutated R2 into negatively charged residue while keeping the first residue unchanged to obtain mutant R2E. According to the solution structure, this mutation would largely change the conformation of R36 and the residues near the loop region by changing the electrostatic environment. This change is shown in the two-dimensional ^1^*H*-^15^*N* HSQC spectra of mutant R2E, in which E27, W26 and other residues close to residue R2, such as R36, L37 and T40, have experienced large chemical shift changes, while the side chains and T40 of residues E27, W26 were far away from residue R2 in the crystal structure, such large structural changes should not happen according to the crystal structure. In general, the two-dimensional ^1^*H*-^15^*N* HSQC spectra of mutant R2E has significantly smaller spectral changes compared with that of 1ΔR2M. Therefore, the huge structural changes caused by 1ΔR2M are more the results of deletion of M1 other than subsidiary changes brought by mutating R2.

**Fig 7:**
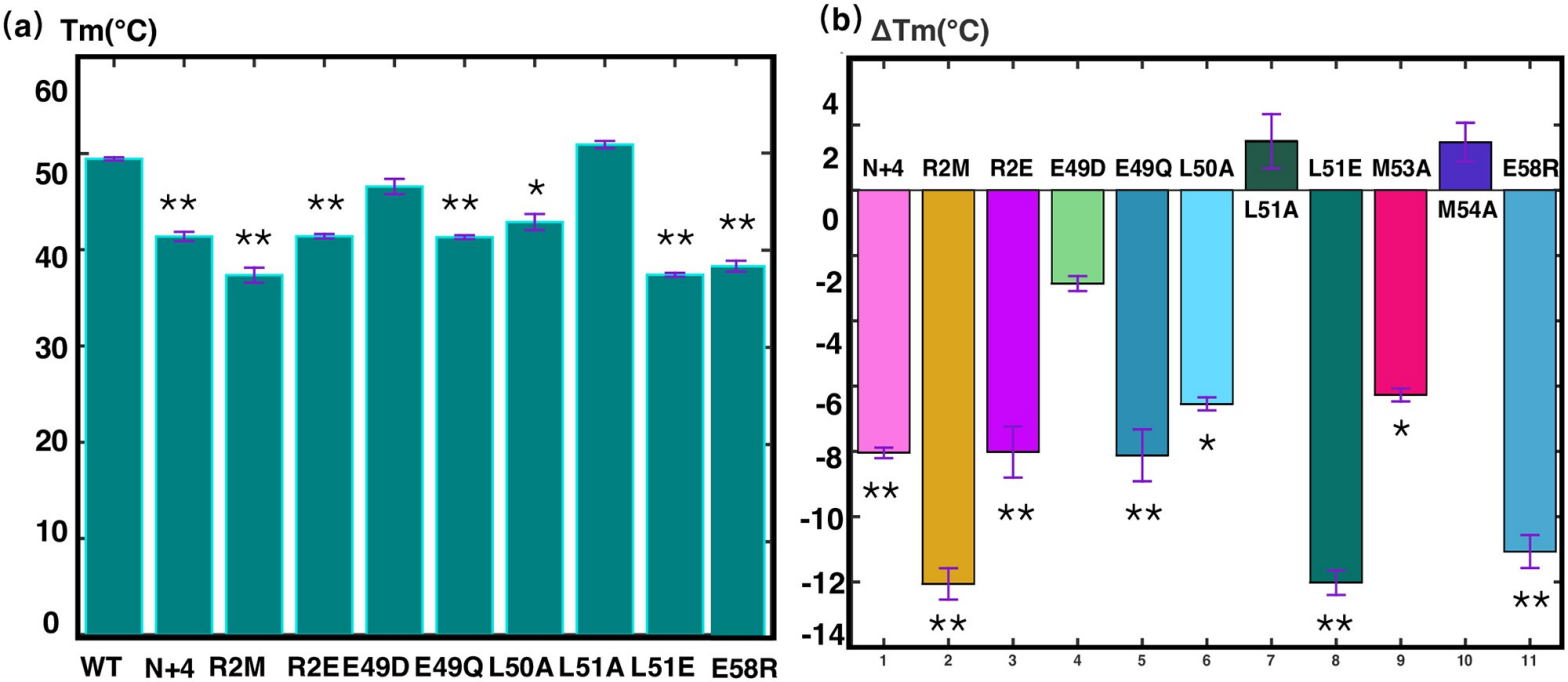
Fluorescence thermal shift assay of PRC1-DD and its mutants (bar graph representation of data in Table 2). **(a)** Bar graph showing the mid-denaturation temperature (Tm) of wild-type PRC1-DD and its mutants. The name of each mutant is labeled on the x-axis of the figure, R2M refers to Δ1R2M. **(b)** Changes in Tm (ΔTm) of mutants compared with wild type. The respective mutants’s colors are as labeled. Graphs were produced in MATLAB, plotted from the mean and standard deviations (thin red T) of Tm from four parallel experiments; single star indicates statistical significance (p<0.05) and double star indicates strong significance (p<0.01).

In addition, we measured the thermal stability of PRC1-DD wild-type and mutants using FTSA, verified the effect of mutations on the protein (Table 2 and Fig 7). FTSA determines the thermal stability of proteins by measuring the midpoint denaturation temperature (Tm) of proteins. We can observe that the Tm values of 1ΔR2M and R2E were significantly lower than those of the wild type, indicating that the thermal stability of the protein was greatly reduced by mutating N terminal residues. A place worth noting is that the Tm value of 1ΔR2M is significantly lower than that of R2E, therefore, we believe that the adverse effects made on the structure of PRC-DD by mutating M1 is not the side effects of mutating residue R2, but further supports the crucial role that M1 plays in stabilizing protein hydrophobic core.

**Table 2:** Tm values of PRC1-DD and its mutants.

Construct	Tm (1)	Tm (2)	Tm (3)	Tm (4)	Average Tm	Stdev
DD WT	49.42	49.23	49.48	49.62	49.44	0.16
DD N+4	41.20	41.60	40.80	41.90	41.38	0.48
DD Δ1R2M	36.36	37.14	37.81	38.13	37.36	0.78
DD R2E	41.20	41.60	41.20	41.60	41.40	0.23
DD E49D	47.20	46.83	46.82	45.40	46.56	0.79
DD E49A	41.20	41.60	41.20	41.20	41.30	0.20
DD L50A	42.20	43.90	43.20	42.20	42.88	0.83
DD L51A	50.40	51.24	50.90	51.15	50.92	0.38
DD L50E	<25	<25	<25	<25	<25	0.00
DD L51E	37.10	37.50	37.50	37.50	37.40	0.20
DD E57A	<25	<25	<25	<25	<25	0.00
DD E58A	37.50	38.70	38.70	38.50	38.35	0.57

#### Residues on Helix H2: L50 and L51

**Fig 8:**
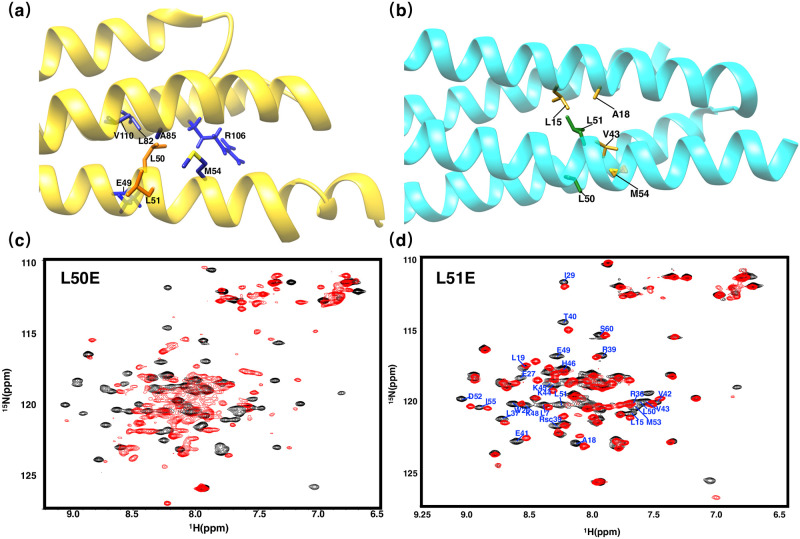
Two-dimensional ^1^*H*-^15^*N* HSQC spectrum of L50E and L51E. Ribbon representation of the **(a)** solution structure (gold) and **(b)** crystal structure (cyan) with the side chains L50 and L51 in orange and green while the side chains of the residues interacting with L50 or L51 shown in blue and mud yellow for solution and crystal structures respectively. **(c)** Two-dimensional ^1^*H*-^15^*N* HSQC spectra of L50E. **(d)** Two-dimensional ^1^*H*-^15^*N* HSQC spectra of L51E, residues experiencing great chemical shift changes (Δ comp ≥ 0.3 ppm) were labeled.

Comparison of solution and crystal structures revealed that residues L50–E58 on Helix H2 exhibit great discrepancies in backbone and side-chain conformations. More specifically, L50 is participating in hydrophobic core formation while L51 locates outside of the protein core according to the solution structure, in contrast, in crystal structure, L51 is the core-forming residue while it is L50 that locates completely outside of the hydrophobic core. To ascertain the conformation of L50 and L51, we mutated either L51 or L50 to E to determine which residue participate in core formation, as residue L50 resides inside the core area according to solution structure, mutating it to an negatively-charged residue will interfere with L50’s electrostatic environment by disrupting its interaction with core residues E49 and R39, but when L51 is mutated to E, little impact should be observed on the overall structure, in contrast, completely opposite effects should be observed according to crystal structure. Shown in Fig 8 are the two-dimensional ^1^*H*-^15^*N*-HSQC spectrum of mutant L50E (red) and L51E (red) superimposed with the wild-type PRC1-DD (black), mutant L50E demonstrated large spectra changes from wild-type, exhibiting structural traits of aggregation and a perturbed hydrophobic core [35, 41], in contrast, the two-dimensional ^1^*H*-^15^*N*-HSQC spectra of mutant L51E showed little overall change compared to wild-type, only residues locating in the vicinity of L51 according to the solution structure showed some chemical shift changes (Fig 8 and S7 Fig). Results from FTSA also demonstrated that L50E is indeed less thermodynamically stable than L51E. In sum, all of above results are consistent with the solution structure (Fig 7).

#### Residues on Helix H2: E57 and E58

**Fig 9:**
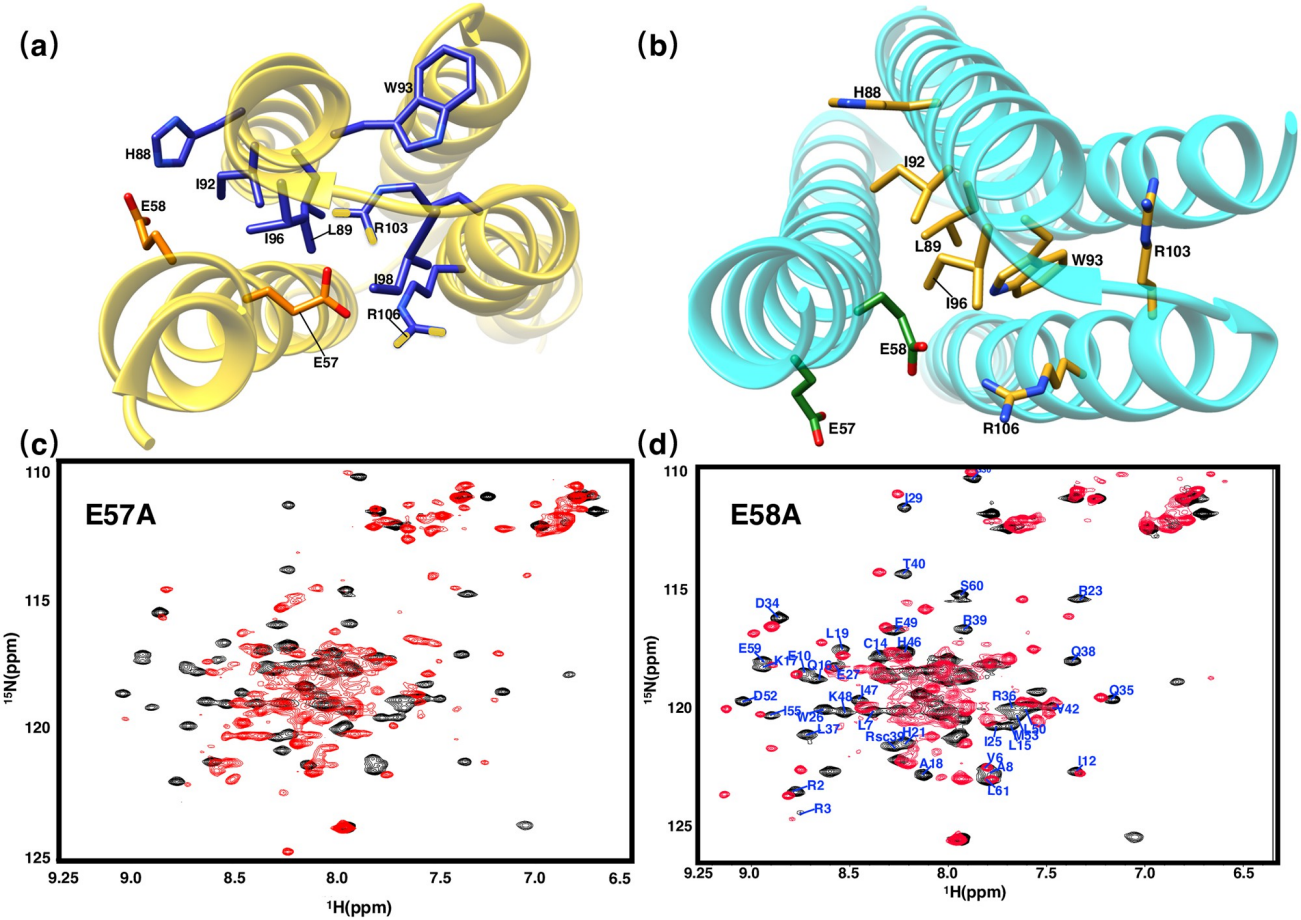
Two-dimensional ^1^*H*-^15^*N* HSQC spectra of E57A and E58A. **(a)-(b)** Ribbon representation of the solution structure (gold) and crystal structure (cyan) with the side chains E57 and E58 in orange and green while the side chains of the residues interacting with E57 or E58 shown in blue and mud yellow for solution and crystal structures respectively. **(c)** Two-dimensional ^1^*H*-^15^*N* HSQC spectra of E57A. **(d)** Two-dimensional ^1^*H*-^15^*N* HSQC spectra of E58A, all residues with significant chemical shift changes (Δ comp ≥ 0.3 ppm) were labeled.

E57, hardly participating in core packing according to crystal structure, plays a key role in the solution structure by forming salt bridges between residues R36 and R39 on the other monomeric unit inside the hydrophobic core; in contrast, based on crystal structure, the salt bridge appears to be between E58 and R39. Here we produced mutants E57A/R and E58A/R to identify the exact position of the salt bridge, mutation E57A/R would abolish the electrostatic interaction between E57, R36 and R39 according to the solution structure, which might also cause a decrease in the stability of protein hydrophobic core, whereas only a small effect on structure should be evident if the conformation of E57 is in agreement with the crystal structure. The respective two-dimensional ^1^*H*-^15^*N*-HSQC spectrum of E57A and E58A are shown in Fig 9. The spectra of E57A is significantly different from wild-type’s and it is characteristic of proteins with a perturbed hydrophobic core, we speculate that mutating E57 disrupted core-stabilizing salt bridges, which might have a substantial impact on overall protein structure, on the other hand, mutant E58A only showed a comparably small change from its original structure (Fig 9(d)). In addition, according to FTSA measurements, the thermal stability of PRC1-DD was more strongly affected by E57 than E58. Therefore, we are justified to believe that solution structure with E57 as the core-stabilizing residue is more consistent with mutational studies (Fig 9).

We have also carefully selected more mutational sites that showed significant differences between solution and crystal structures with above mentioned criterion, such as M53, M54 and E49 (S8 and S9 Figs) etc. and other mutational results are presented in Supporting information.

### Assessing the precise conformations of full-length PRC1

**Fig 10:**
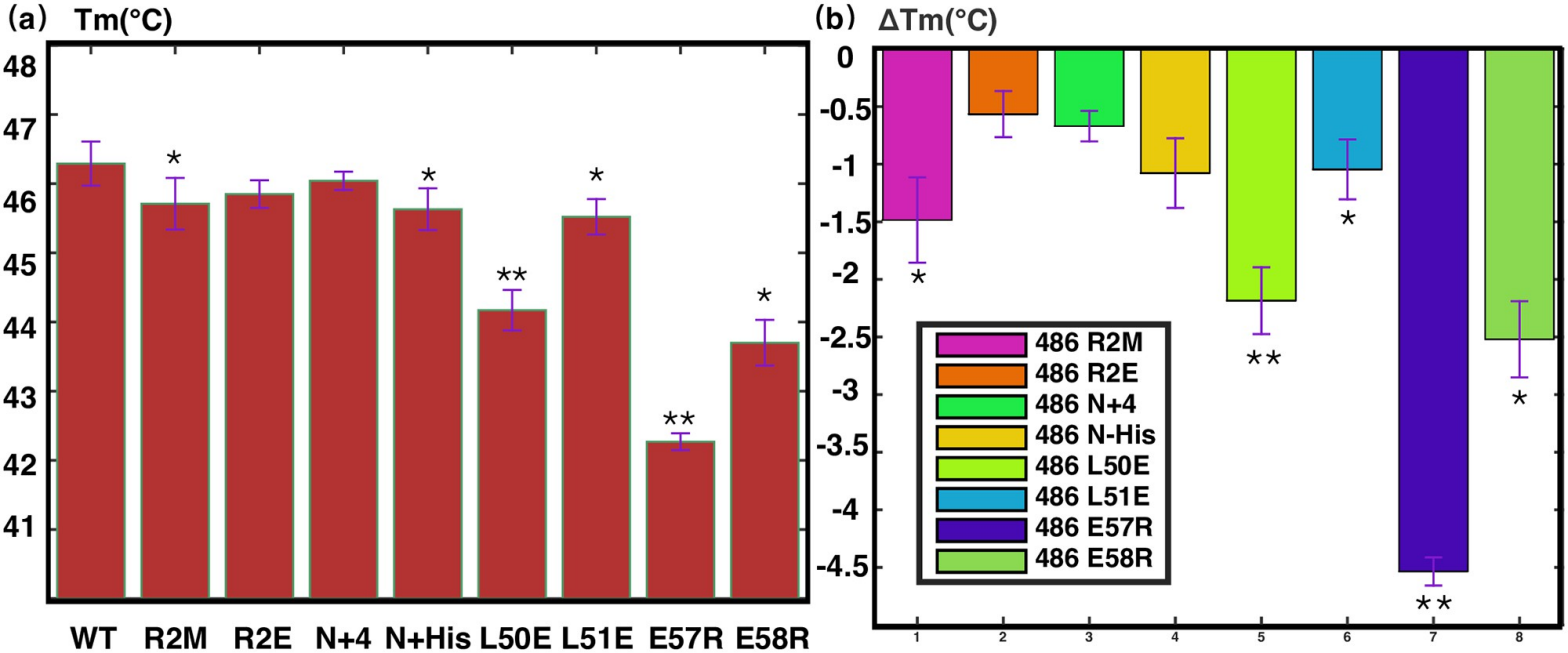
Results from fluorescence thermal shift assay (FTSA) of PRC1 1–486 and its mutants (bar graph representation of data in Table 3). **(a)** Bar graph showing the mid-denaturation temperature (Tm) of wild-type PRC1 1–486 and its mutants. The name of each mutant is labeled on the x-axis of the figure, R2M refers to Δ1R2M. **(b)** Changes in Tm (ΔTm) of mutants compared with wild type. The mutants are color-coded according to the figure legend. Graphs were produced in MATLAB; the mean and standard deviation of Tm were calculated from four parallel runs of each experiment. single star indicates statistical significance (p<0.05) and double star indicates strong significance (p<0.01).

Even though PRC1-DD is an independent protein domain in terms of structure and function, it is nonetheless a truncated segment of the full-length protein; therefore, the question of whether the full-length protein possesses the same structural properties of PRC1-DD still remained. To address this problem, we engineered mutations R2M, R2E, N+4, N+His, L50E, L51E, E57R and E58R, into PRC1 1–486 (aa: 1–486) which represents the full-length PRC1. The selected mutations were proven to be most characteristic of the structural discrepancies between the solution and crystal structures. The thermal stability of PRC1 1–486 wild-type and its mutants were measured by FTSA (Fig 10 and Table 3). Agreement can be observed on the pattern of protein stability between mutants on PRC1 1–486 and that of PRC1-DD. For instance, N-terminus mutants PRC1 1–486ΔR2M, PRC1 1–486-R2E, PRC1 1–486-N+4 and PRC1 1–486-N+His affect the stability of PRC1 1–486 in the same way as corresponding mutations on PRC1-DD, even though on a lesser extent. Mutant pairs L50E and L51E, E57R and E58R on PRC1 1–486 reproduced the same thermal stability pattern on PRC1-DD, with PRC1 1–486-L50E and PRC1 1–486-E57R being less thermostatically stable than PRC1 1–486-L51E and PRC1 1–486-E58R, respectively, accentuating the crucial roles residues L50 and E57 partake in full-length PRC1, which is only consistent with the solution structure. In sum, all of the above mutational studies demonstrated that PRC1-DD and full-length PRC1 showed a strong possibility of possessing the same structural properties, excluding the likelihood that the structural discrepancies between solution and crystal structures were a consequence of protein truncation.

**Table 3:** Tm values of PRC1 1–486 and its mutants.

Construct	Tm (1)	Tm (2)	Tm (3)	Tm (4)	Average Tm	Stdev
1_486 WT	46.29	46.51	46.75	47.03	46.65	0.32
1_486 Δ1R2M	45.71	44.95	45.07	44.91	45.16	0.37
1_486 R2E	44.96	45.46	45.46	45.69	45.39	0.31
1_486 N+4	46.04	45.82	46.12	45.92	45.98	0.13
1_486 N-His	45.63	45.96	45.42	45.26	45.57	0.30
1_486 L50E	44.17	44.77	44.64	44.25	44.46	0.29
1_486 L51E	45.52	45.89	45.29	45.7	45.60	0.26
1_486 M53A	44.25	44.77	44.81	43.84	44.42	0.46
1_486 M54A	46.87	46.75	47.04	46.84	46.88	0.12
1_486 E57R	42.27	42.01	42.14	42.02	42.11	0.12
1_486 E58R	43.7	44.2	44.5	44.1	44.13	0.33

## Discussion

By comparing the solution and crystal structures, we found that they differ significantly in folding and hydrophobic core composition. The H/D exchange dynamics of each residue in PRC1-DD and the mutational studies revealed that the solution structure better reflects the conformation PRC1 adopted in solution condition. Here, we will discuss the implications of the results shown in the results section and explain the possible reasons for the differences between the two structures.

Close inspection of the crystal structure showed that there exist a greater than average crystalline B-factors (S11 Fig) in PRC1 N terminus and in the end of helix H2, which is a measure of structural uncertainty induced by protein dynamics and thermal disorder [42, 43]. The different conformations observed under solution and crystal conditions provided further support for the inherent dynamics of residues in the above-mentioned sites [44]. The solution structure, together with previously reported Electron Microscopy analysis which lack Cryo-EM density in the dimerization domain [13, 14], suggest that PRC1 dimerization domain is an inherently dynamic structure, and may possibly adopt different conformations in different conditions.

**Fig 11:**
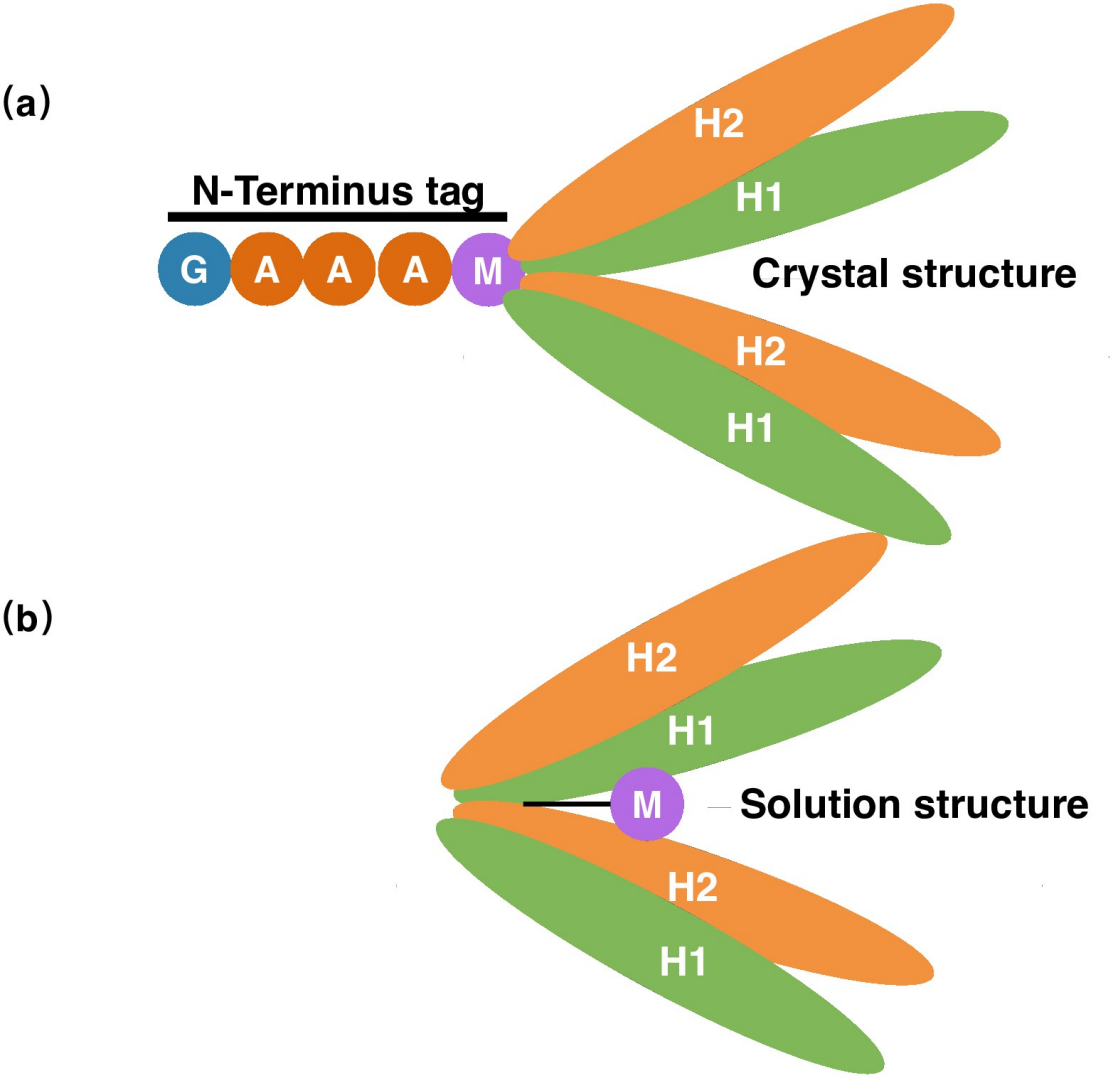
Schematic diagram of full-length PRC1 (a) crystal and (b) solution structure.

**Fig 12:**
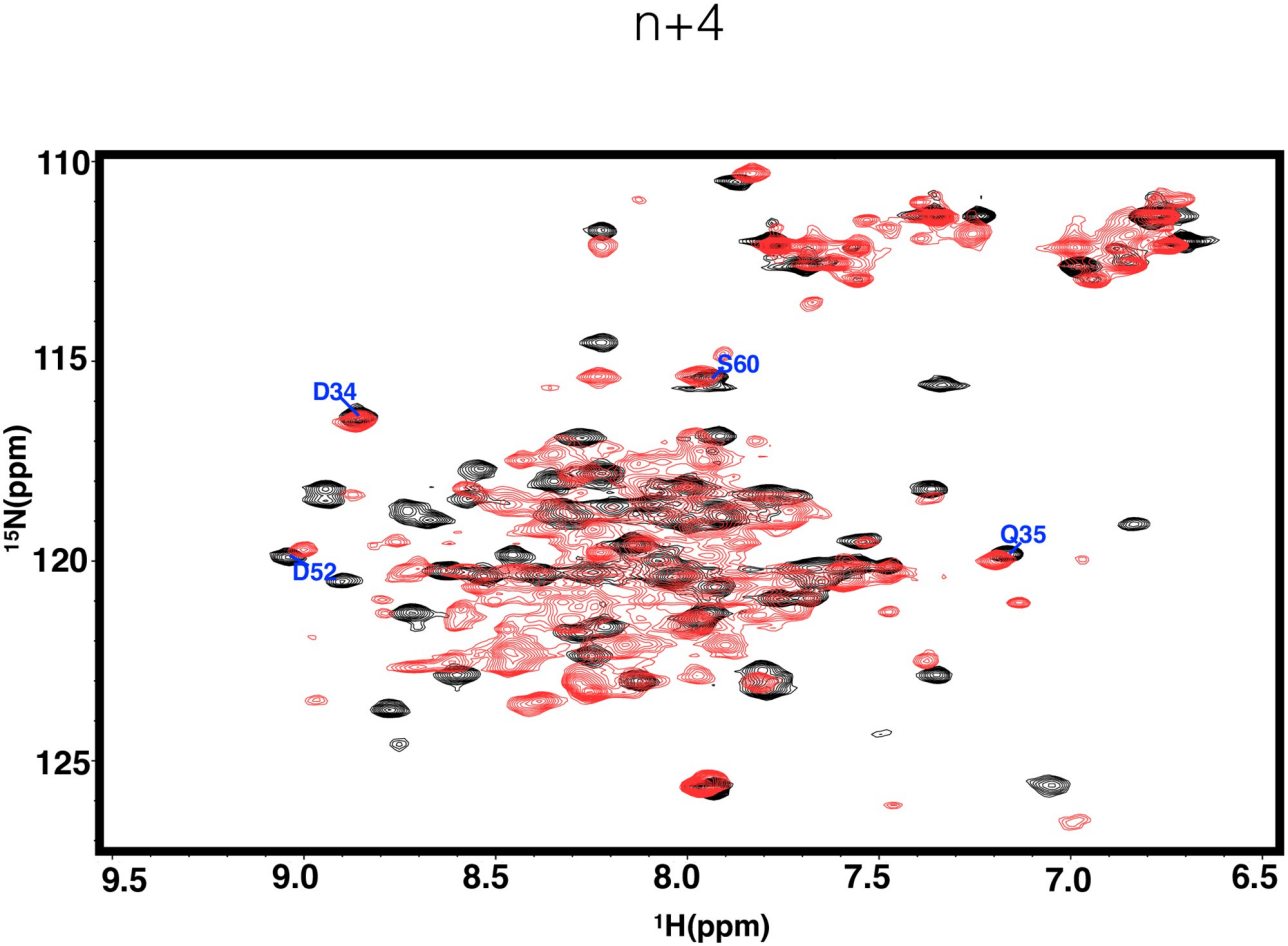
Two-dimensional ^1^*H*-^15^*N* HSQC spectra of N terminal extension mutant (red) and wildtype (black).

The N terminus of the protein possess a unique structural trait, with residue M1 inserting into the hydrophobic core. In general, a protein’s N terminal residues should be flexible and stretched out of the hydrophobic core, insignificant to the overall hydrophobic packing of the protein, but for others like SARS-CoV Main protease (Mpro), BFX, FHV coat protein etc., the first residue is indispensable for the protein’s function or structural integrity, and extra residues attached to the N terminus of such proteins will disturb the correct conformation of its overall protein structure, abolishing its biological function, or even inducing protein degradation. In the solution structure of PRC1-DD, residue M1 is inserted into protein hydrophobic core to maintain the overall protein structural integrity, while M1 is outside of the protein core according to the crystal structure. Careful inspection of the PRC1 construct used for crystallization revealed that an additional sequence of residues 'GAAA'was added to the N-terminus of PRC1 protein. It is likely that this additional tag prevented M1 from being successfully inserted into the hydrophobic core due to spatial constraints (Fig 11). Thus, to determine whether the N-terminal tag does affect the structure of PRC1, we added the sequence 'GAAA'and His tags to the N terminus of PRC1-DD and PRC1 1–486, creating mutants PRC1-DD-N+4, PRC1-DD-N+His and PRC1–486-N+4, PRC1–486-N+His. The protein expression of PRC1-DD-N+4 and PRC1-DD-N+His were significantly lower than that of wild type (S10 Fig), and the thermal stability of N terminal extended mutants are also significantly lower than that of the wild type (Fig 7). The structure of PRC1-DD, was also significantly affected by N terminal extensions, even producing an aggregatory (Fig 12) effect on the protein. Protein aggregation is the result of improper folding, suggesting that N terminal residues may even participate in the folding process of the protein. Furthermore, for full-length PRC1 protein, mutants PRC1 1–486-N+4 and PRC1 1–486-N+His both showed great decreases in thermal stability compared to wild-type PRC1 1–486 (Fig 10), indicating that the addition of tag GAAA or 6His at the N-end of the protein leave huge impacts on the stability and possibly the structural integrity of the whole protein.

The structural significance of the special N terminus conformation of PRC1 might be that the core placing N terminal residues attribute a more compact and globular shape to the protein, incorporating more residues into the hydrophobic surface, created a larger and stronger hydrophobic core, more efficiently stabilizing the protein.

## Conclusion

In this study, we determined the structure of the N-terminal dimerization domain. We further characterized the differences between the solution and the crystal structure, we found that the N terminus, loop region and helix H2 exhibit great differences in the solution structure and crystal structure, which directly leads to the differences in the composition of the residues that make up the hydrophobic core of the solution structure and crystal structure. We also verified the validity of the the solution structure relative to the crystal structure, speculated the correct dimerization mechanism of PRC1 under solution conditions, and corrected the mistakes in previous studies. Furthermore, we presented explanations for the differences in the solution structure and crystal structure.

Then, we verified the solution structure of PRC1-DD by H/D exchange experiments and mutational studies. In the H/D exchange experiments, the residues located in the hydrophobic core of the solution structure, such as L19, L50, M53 and E57, possess higher protection factors than residues A18, L51, M54 and E58 which are speculated to be located in the hydrophobic core according to the crystal structure, these results verifies the reliability of the solution structure relative to the crystal structure. In the mutational studies, mutations 1ΔR2M, L50 (L50A) and E57 (E57A) should have great impacts on the structural integrity and thermal stability of the protein core according to the solution structure, but according to the crystal structure, these residues should have little impact on the structure or the stability of the protein. We tracked the changes in structure and thermal stability of each mutant through two-dimensional ^1^*H*-^15^*N*-HSQC spectrum and FTSA. mutation. The large structural changes and reduced thermal stability caused by 1ΔR2M, L50E/A and E57A verified that the structural characteristics of PRC1-DD in solution are more in line with our solution structure. Similarly, we generated the above mutations on full-length PRC1 (PRC1 1–486). We found the mutations on PRC1 1–486 reproduced the same pattern of structural stability as PRC1-DD, therefore, we strongly speculate that the structural characteristics of the PRC1-DD solution structure are also present on full-length protein. In sum, we conclude the solution structure better describes the structure of PRC1 in the dimerization domain.

Finally, in the construct used for crystal structure determination, the additional four residues GAAA attached at its N terminus may prevented residue M1 from inserting into the hydrophobic core due to spatial constraints, and affected the correct folding of the hydrophobic core of the protein. Therefore, we believe that the reason why the structure of PRC1-DD in solution is different from that in crystallization is the addition of the N-terminal redundant sequence in the crystal structure.

## Supporting information

S1 Fig PRC1-DD in high performance liquid chromatography. (a) PRC1-DD in different concentration of urea. (b) PRC1-DD under different temperatures. (c) PRC1-DD at different concentrations.
(PDF)

S2 Fig Assessing the molecular weight of PRC1-DD. (a) Static Light Scattering spectrum of PRC1-DD. (b) Chemical cross-linking result of PRC1-DD, in which lane 1 has no cross-linker added, lane 2 is the state of PRC1-DD with cross-linker.
(PDF)

S3 Fig Two-dimensional ^1^*H*-^15^*N* HSQC spectra of PRC1-DD under different conditions. (a) PRC1-DD (black) compared with itself after 7 days(blue). (b) PRC1-DD under room temperature (black), 25°C(blue) and 35°C (green). (c) PRC1-DD at 0.5mM (black) compared with itself at 0.1mM (red). (d) enlargement of the box in (c). (e) PRC1-DD (black) and with 6M urea (red). (f) PRC1-DD (black) and with 8M urea (red).
(PDF)

S4 Fig The RMSD values of PRC-DD solution and crystal structures under different alignment conditions. (a) aligning a single monomeric unit. (b) aligning the whole homodimeric structures of PRC1-DD. (c) aligning a single monomeric unit in the homodimer. Residue number 1–67 denote the first monomeric unit, while 67–134 denote the second monomeric unit.
(PDF)

S5 Fig Slices from three-dimensional ^1^*H*-^13^*C* NOESY-HSQC spectrum of PRC1-DD, that show characteristic cross-peaks from residues that possess distinctive conformations in solution and crystal structures. (PDF)

S6 Fig Structural variation of PRC1-DD wild-type and mutants mapped to structure, residue R2 is represented in stick representation. (a) For mutant Δ1R2M, residues with great chemical shift changes compared to wild-type are mapped to PRC1-DD structure. (b) For mutant R2E, areas with great chemical shift changes are mapped to PRC1-DD structure. Red denote areas with chemical shift change of 8 ppm or more, white denotes chemical shift change of around 5 ppm, blue shows no chemical shift change.
(PDF)

S7 Fig Structural variation of PRC1-DD wildtype and mutants mapped onto structure. (a) For mutant L51E, complex chemical shift changes (Δ*δ* comp) of amide hydrogen ^1^*H* and nitrogen ^15^*N* were calculated and shown as bar graph. (b) Areas with significant complex chemical shift changes induced by mutation L51E are mapped onto PRC1-DD structure. Red denote areas with chemical shift change of 8ppm or more, white denote chemical shift change of around 5 ppm, blue shows no chemical shift change.
(PDF)

S8 Fig Two-dimensional ^1^*H*-^15^*N* HSQC spectrum of E49D and E49A compared with wildtype PRC1-DD. (a) Ribbon representation of solution structure, in which residues E49, H46 and those residues that interact with it were stressed in ball-and-stick representation. (b) crystal structure of PRC1-DD, in which residues E49, H46 and those residues that interact with it were stressed in ball-and-stick representation. (c) The superposition of two-dimensional ^1^*H*-^15^*N* HSQC spectrum of wildtype(black) and E49D(red), in which residues exhibiting large changes are denoted. (d) The superposition of two-dimensional ^1^*H*-^15^*N* HSQC spectrum of wildtype(black) and E49A(red), in which residues exhibiting large changes are denoted.
(DOCX)

S9 Fig Long-range two-dimensional ^1^*H*-^15^*N* HSQC showing PRC1-DD imidazole, in which black denotes wildtype, red denotes E49D, green denotes E49A. (DOCX)

S10 Fig SDS-PAGE gel showing the expression levels of PRC1-DD WT, mutant PRC1-DD-N+4 and PRC1-DD-N+His. Each lane shows the eluted protein profile with induction time labeled on top of each lane. The expression system in E.*Coli*/*Rosseta* (DE3). The molecular weight of PRC1-DD is around 15kD.
(PDF)

S11 Fig Crystal structure B-factors of each residue, values shown here were taken from PDB entry 4L6Y. (PDF)

S1 Table Random-coil factor *k*_*rc*_ for every residue pair in PRC1-DD. (DOCX)

S2 Table SASA values of solution and crystal structures. (DOCX)

S3 Table Distances of atom from residues that have variations in conformation between solution and crystal structure. (DOCX)

S4 Table Table showing the primer sequences of wild-type and mutants (F is for forward primer, R is for reverse primer, restriction enzyme sites were indicated in the name of each primer). (DOCX)

S5 Table Table showing residual dipolar coupling (DD) from PALES. (DOCX)

S6 Table Table showing RDC alignment tensors of PRC1-DD (generated by software PALES). (DOCX)

S7 Table Table showing the SAXS data processed by different softwares, the SAXS data was collected with protein concentration of 10mg/ml for 8h, PV is for porod volume. (DOCX)
