# Supplementary figures and images for: Validation of the solution structure of dimerization domain of PRC1
Source: PLoS One. 2022 Aug 5;17(8):e0270572. doi: 10.1371/journal.pone.0270572 (PMC9355583; doi:10.1371/journal.pone.0270572)

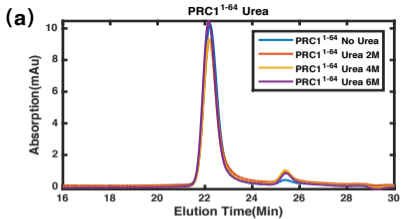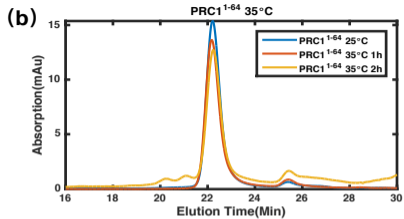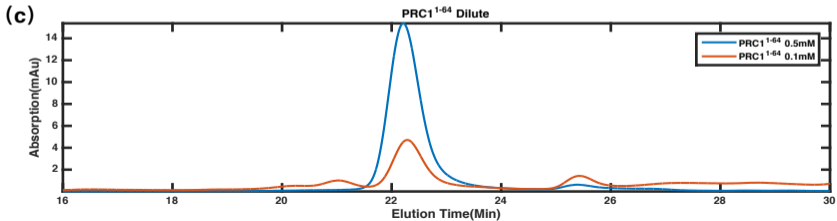

Supplement: S1 Fig — (a) PRC1-DD in different concentration of urea. (b) PRC1-DD under different temperatures. (c) PRC1-DD at different concentrations. (PDF) [file pone.0270572.s001.pdf]

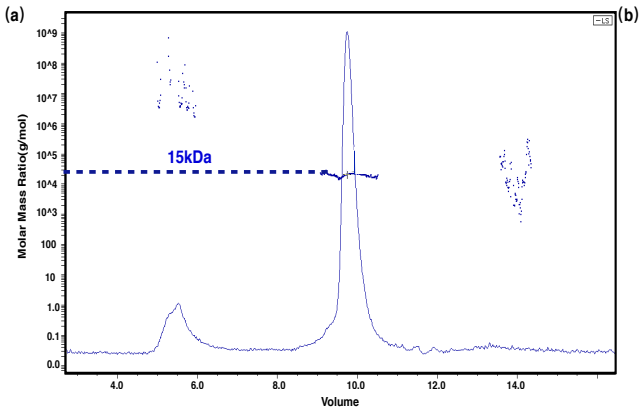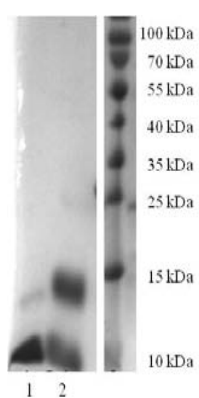

Supplement: S2 Fig — (a) Static Light Scattering spectrum of PRC1-DD. (b) Chemical cross-linking result of PRC1-DD, in which lane 1 has no cross-linker added, lane 2 is the state of PRC1-DD with cross-linker. (PDF) [file pone.0270572.s002.pdf]

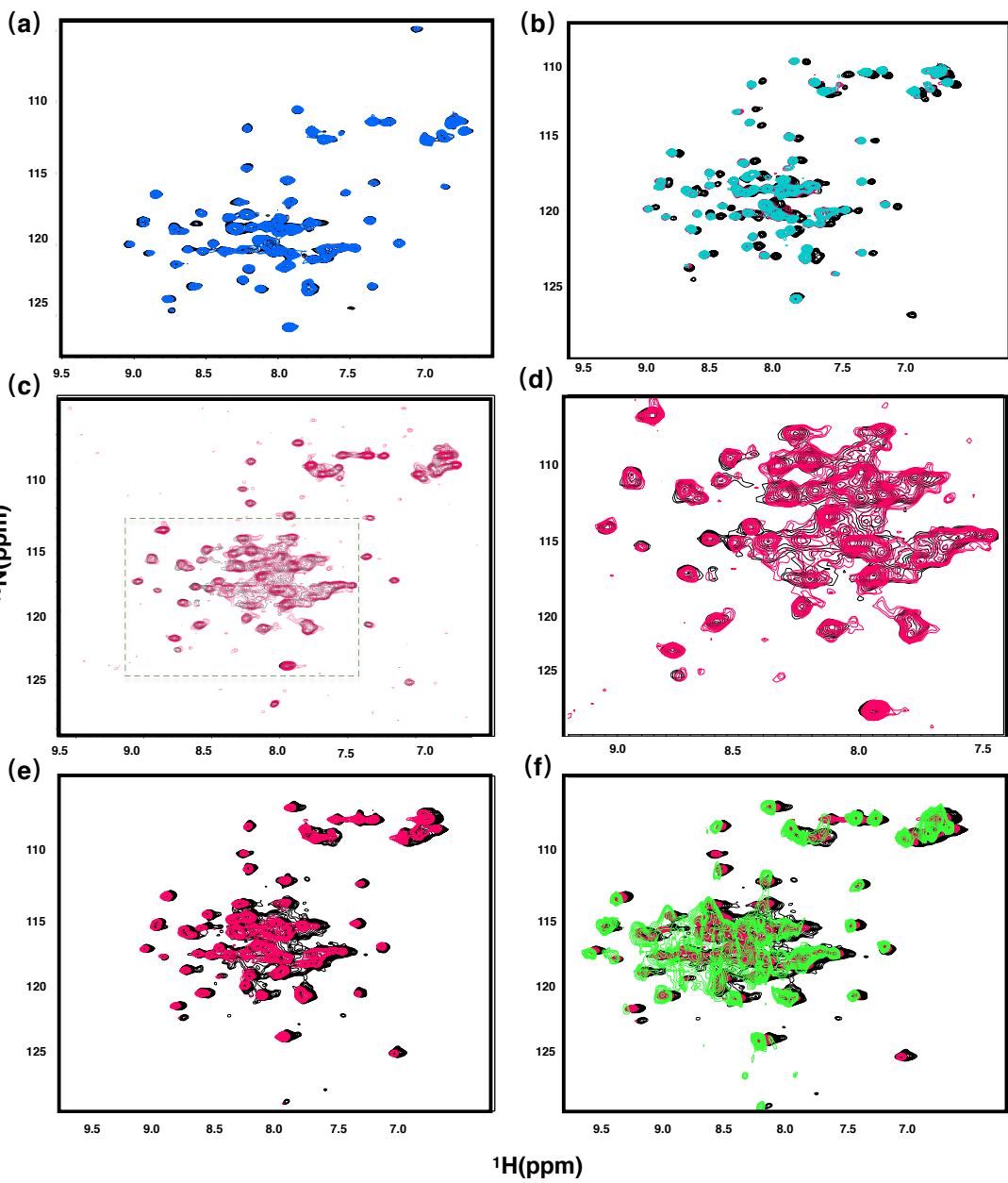

Supplement: S3 Fig — (a) PRC1-DD (black) compared with itself after 7 days(blue). (b) PRC1-DD under room temperature (black), 25°C(blue) and 35°C (green). (c) PRC1-DD at 0.5mM (black) compared with itself at 0.1mM (red). (d) enlargement of the box in (c). (e) PRC1-DD (black) and with 6M urea (red). (f) PRC1-DD (black) and with 8M urea (red). (PDF) [file pone.0270572.s003.pdf]

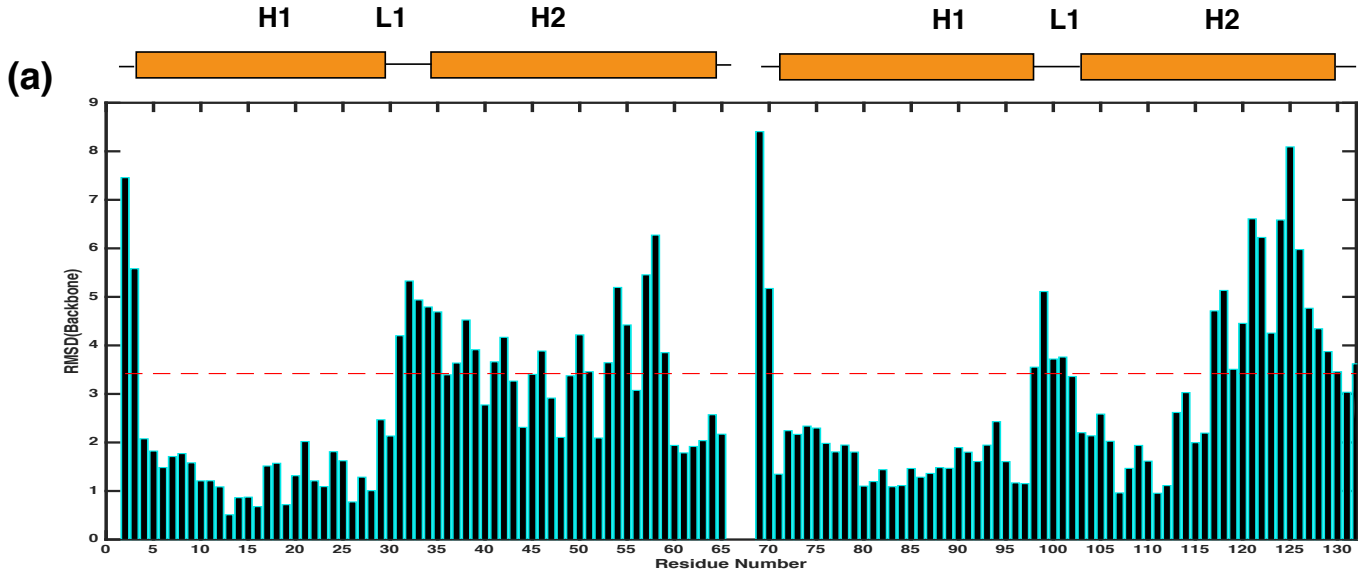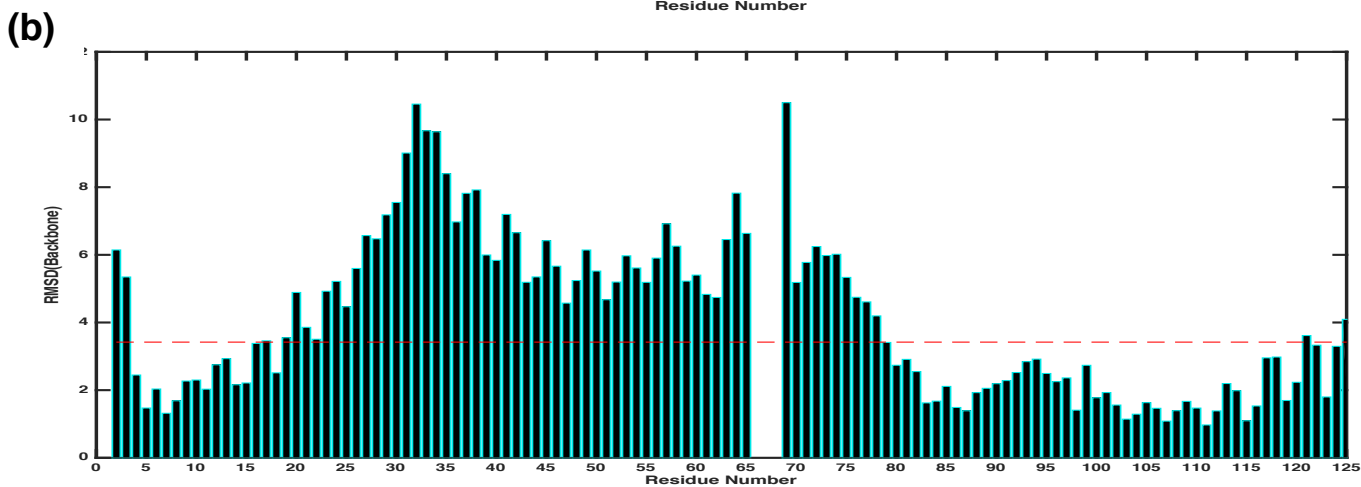

Supplement: S4 Fig — (a) aligning a single monomeric unit. (b) aligning the whole homodimeric structures of PRC1-DD. (c) aligning a single monomeric unit in the homodimer. Residue number 1–67 denote the first monomeric unit, while 67–134 denote the second monomeric unit. (PDF) [file pone.0270572.s004.pdf]

MET1CB

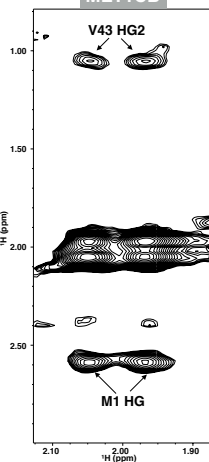

MET1CE

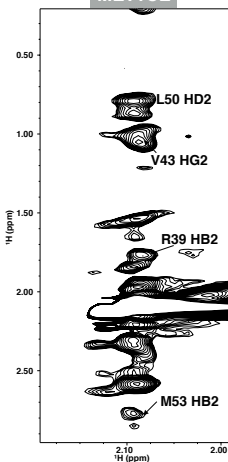

MET1CE

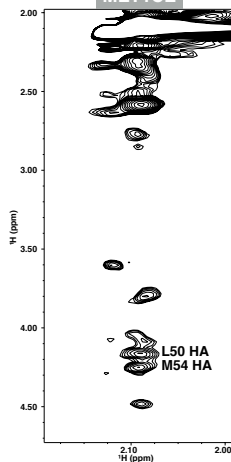

Leu50CD1

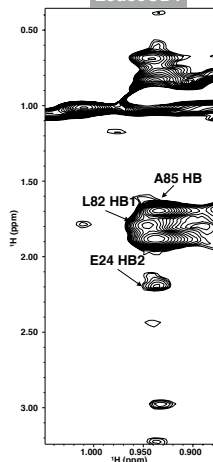

Leu50CD2

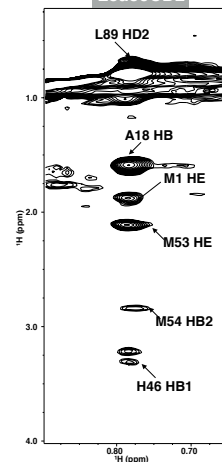

MET53CE

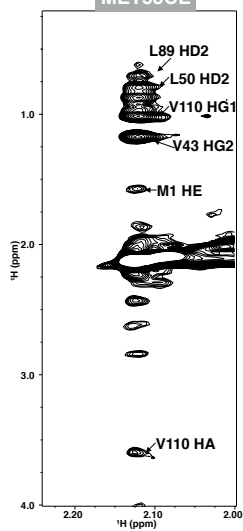

MET54CE

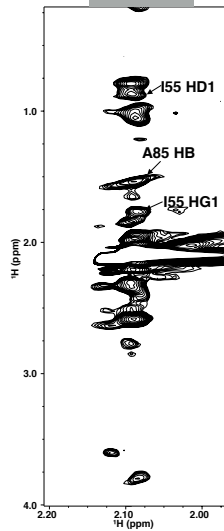

GLU57CA

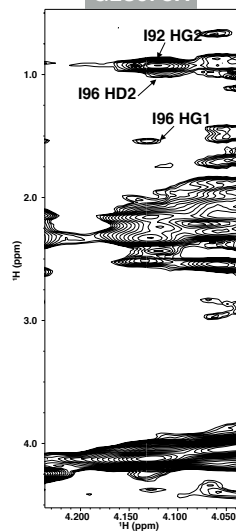

GLU57CB

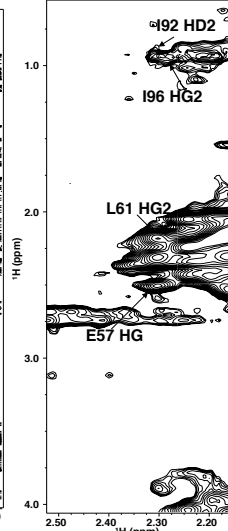

GLU57CB

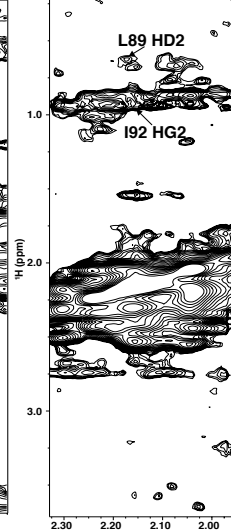

Supplement: S5 Fig — (PDF) [file pone.0270572.s005.pdf]

**(a)**

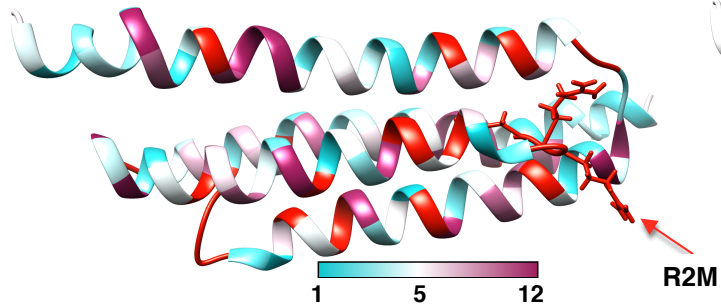

**(b)**

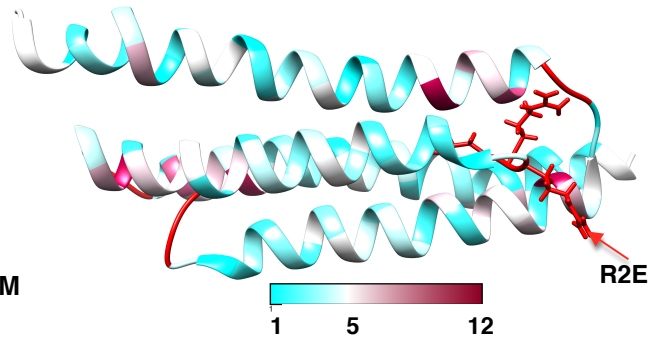

Supplement: S6 Fig — (a) For mutant Δ1R2M, residues with great chemical shift changes compared to wild-type are mapped to PRC1-DD structure. (b) For mutant R2E, areas with great chemical shift changes are mapped to PRC1-DD structure. Red denote areas with chemical shift change of 8 ppm or more, white denotes chemical shift change of around 5 ppm, blue shows no chemical shift change. (PDF) [file pone.0270572.s006.pdf]

**(a)**

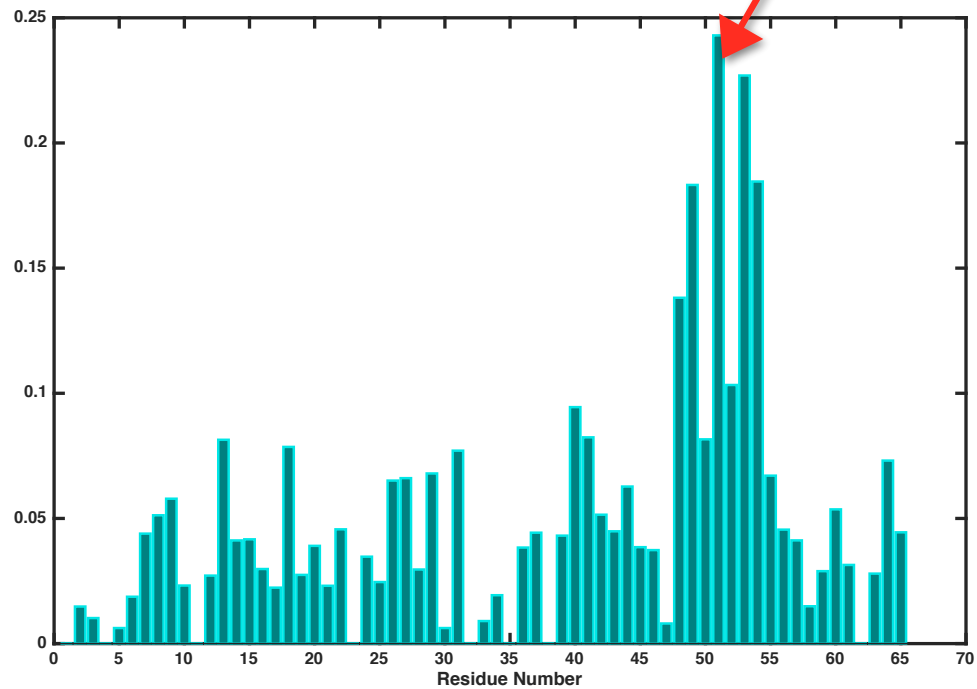

**(b)**

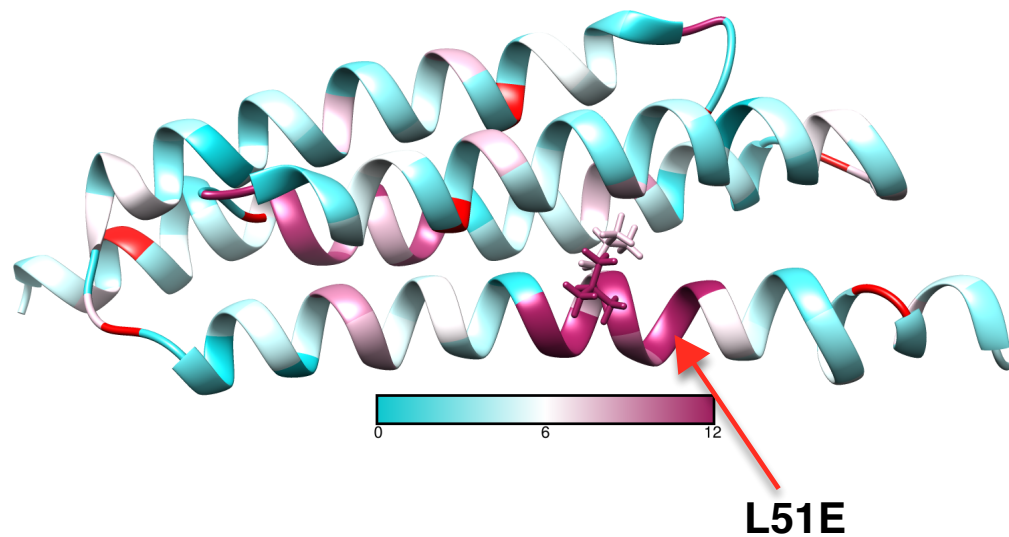

Supplement: S7 Fig — (a) For mutant L51E, complex chemical shift changes (Δδ comp) of amide hydrogen 1H and nitrogen 15N were calculated and shown as bar graph. (b) Areas with significant complex chemical shift changes induced by mutation L51E are mapped onto PRC1-DD structure. Red denote areas with chemical shift change of 8ppm or more, white denote chemical shift change of around 5 ppm, blue shows no chemical shift change. (PDF) [file pone.0270572.s007.pdf]

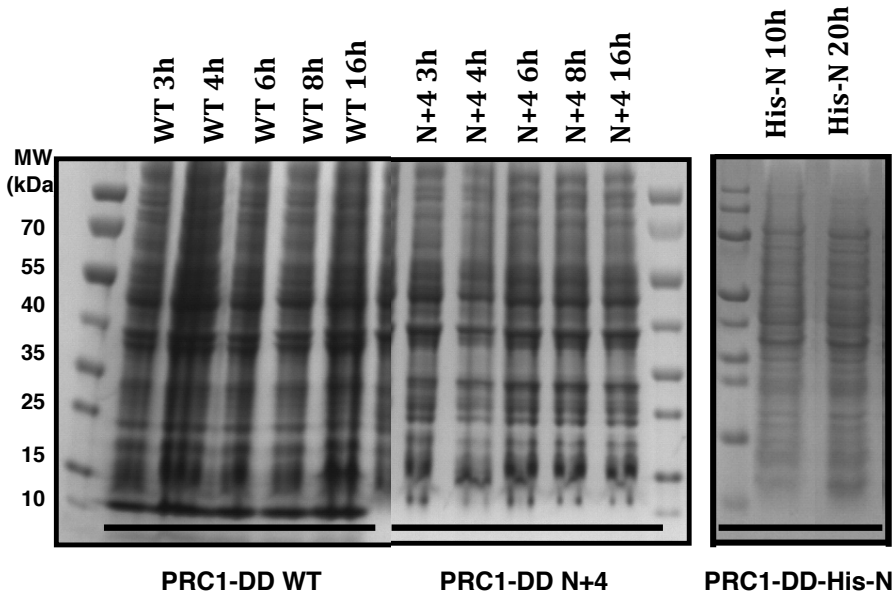

Supplement: S10 Fig — Each lane shows the eluted protein profile with induction time labeled on top of each lane. The expression system in E.Coli/Rosseta (DE3). The molecular weight of PRC1-DD is around 15kD. (PDF) [file pone.0270572.s010.pdf]

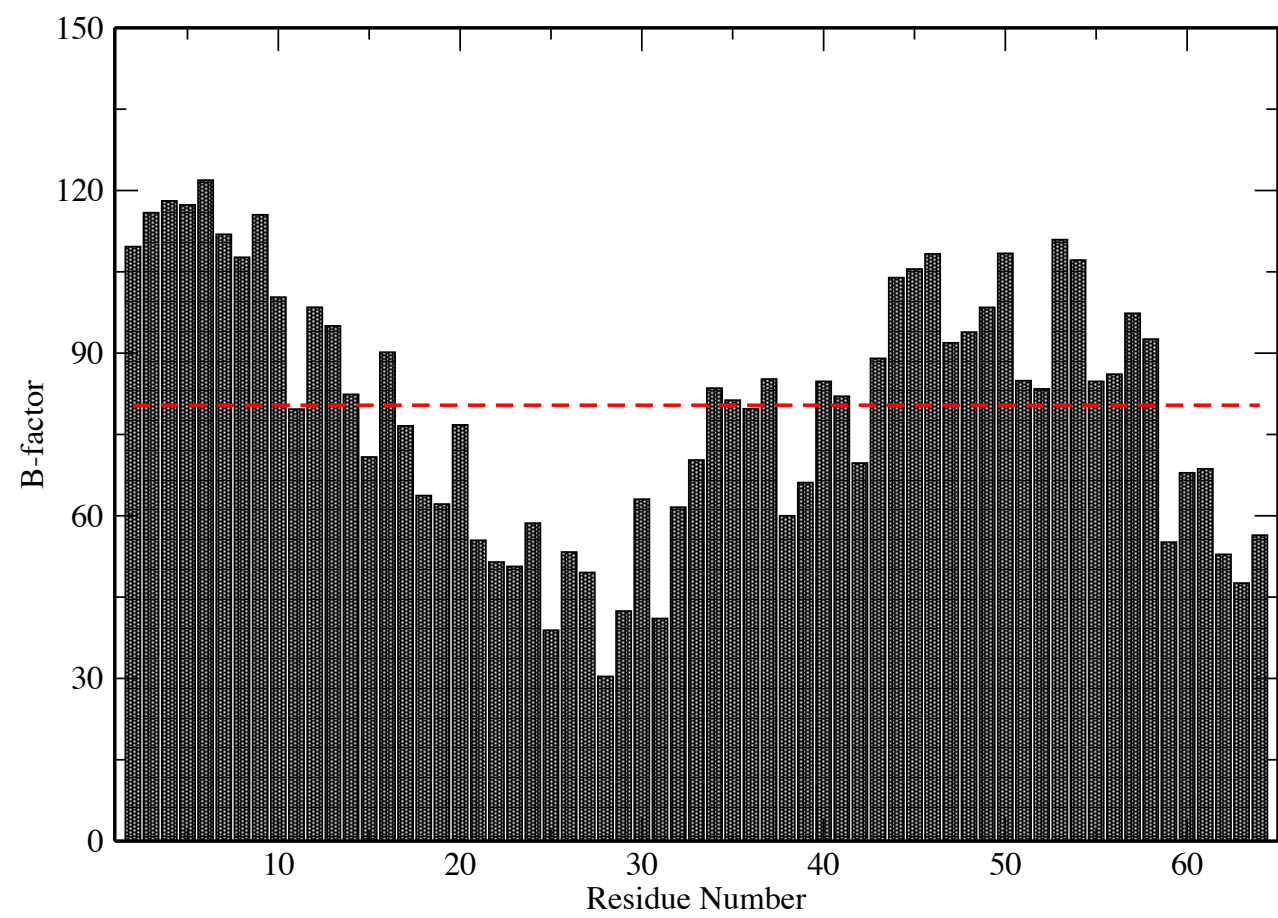

Supplement: S11 Fig — (PDF) [file pone.0270572.s011.pdf]
