# Supplementary material for: Validation of the solution structure of dimerization domain of PRC1
Source: PLoS One. 2022 Aug 5;17(8):e0270572. doi: 10.1371/journal.pone.0270572 (PMC9355583; doi:10.1371/journal.pone.0270572)
Supplement: S8 Fig — (a) Ribbon representation of solution structure, in which residues E49, H46 and those residues that interact with it were stressed in ball-and-stick representation. (b) crystal structure of PRC1-DD, in which residues E49, H46 and those residues that interact with it were stressed in ball-and-stick representation. (c) The superposition of two-dimensional 1H-15N HSQC spectrum of wildtype(black) and E49D(red), in which residues exhibiting large changes are denoted. (d) The superposition of two-dimensional 1H-15N HSQC spectrum of wildtype(black) and E49A(red), in which residues exhibiting large changes are denoted. (DOCX) [file pone.0270572.s008.docx]

The conformation of residue E49 on α-helix H2 varies greatly between solution structure and crystal structures. The ^15^N_ξ2_ on the imidazole ring of H46 forms a hydrogen bond with the carboxyl of E49. In the solution structure, the hydrogen bonds between the two include intramolecular hydrogen bonds and intermolecular bonds. On the contrary, in the crystal structure, the spatial distance between H46 and E49 is 10 Å, which does not meet the condition for intermolecular hydrogen bond formation, as shown in S8 Fig (a)(b).

The validity of solution structure can be further verified by validating the existence of intermolecular hydrogen bond between H46 and E49. Mutating E49 into E49D and E49A increases the distance between the carboxyl of residue E49 and the imidazole ring of H46, weakens (E49D) or destroys (E49A) intramolecular and intermolecular hydrogen bonds, and detract the stability of the dimerization interface. Imidazole nitrogens ^15^N_ξ2_, ^15^N_δ1_ and imidazole hydrogens ^1^Hξ1,^1^H_δ2_ can be observed in long-range 2D ^1^H-^15^N-HSQC spectrum. There is a correlation between the chemical shift of the atom on the imidazole ring and the stability of the hydrogen bond that the atom partakes.

To determine the state of the hydrogen bond between H46 and E49, We used long-range 2D ^1^H-^15^N-HSQC spectra to assess the correlations between the atoms of ^15^N_ξ2_ and ^1^H_ξ1_，^15^N_δ1_ and ^1^H_δ2_ on imidazole ring of both the PRC1-DD wildtype, mutant H49E and H49A. The chemical shift of ^15^N_ξ2_ and ^15^N_δ1_ is sensitive to the protonation state of imidazole ring and the stability of its hydrogen bond[1-3].

According to the literature, the chemical shift of acceptor of hydrogen bond usually moves to downfield, while the donor in hydrogen bond moves upfield[4]. In mutants E49D and E49A, the chemical shift of ^15^N_ξ2_ on 2D ^1^H-^15^N-HSQC spectrum moves to lower chemical shift (upfield), while the chemical shift of ^15^N_δ1_ moves to the direction of higher chemical shift (downfield). This change in the chemical shift of imidazole ^15^N_ξ2_ and ^15^N_δ1_ reflects the microenvironment change of H46 imidazole caused by mutation. In which, mutation E49D weakens the stability of hydrogen bond between the carboxyl of residue E49 and the imidazole of residue H46, while mutation E49A destroys the hydrogen bond. As shown in S8 Fig (b), mutation E49D caused the chemical shift change of ^15^N_δ1_ and ^15^N_ξ2_ relative to that of wild type, while in mutant E49A, the chemical shift changes of ^15^N_δ1_ and ^15^N_ξ2_ deviated more from the wildtype than that of mutant E49D. These result shows that there is indeed a hydrogen bond between E49 and H46 (S8 Fig.).

**S8 Fig.** 2D ^1^H-^15^N HSQC spectrum of E49D and E49A compared with wildtype PRC1-DD.

**(a)** Ribbon representation of solution structure, in which residues E49, H46 and its interactors were stressed in ball-and-stick representation. **(b)** crystal structure of PRC1-DD, in which residues E49, H46 and its interactors were stressed in ball-and-stick representation. **(c)** The superposition of 2D ^1^H-^15^N HSQC spectrum of wildtype(black) and E49D(red), in which residues exhibiting large changes are denoted. **(d)** The superposition of 2D ^1^H-^15^N HSQC spectrum of wildtype(black) and E49A(red), in which residues exhibiting large changes are denoted.
