# Supplementary material for: Validation of the solution structure of dimerization domain of PRC1
Source: PLoS One. 2022 Aug 5;17(8):e0270572. doi: 10.1371/journal.pone.0270572 (PMC9355583; doi:10.1371/journal.pone.0270572)
Supplement: S9 Fig — (DOCX) [file pone.0270572.s009.docx]

In order to verify whether there are intermolecular hydrogen bonds between E49 and h46 at the same time, we used FTSA to detect the difference between the thermal stability of E49D and E49A and the wild type (Fig 7. in the main text). Compared with the wild type, the midpoint denaturation temperature (Tm) of E49A decreased significantly (> = 3°C), indicating that residue E49 stabilizes the dimerization interface, and E49 participates in the formation of intermolecular salt bridge(because in the case of only intramolecular hydrogen bond formation, no change in thermal stability should be observed between E49D and E49A. In conclusion, we believe that E49 participates in the formation of intermolecular hydrogen bond, which is consistent with the solution structure of PRC1-DD.

**S9 Fig.** Long-range 2D ^1^H-^15^N HSQC showing PRC1-DD imidazole, in which black denotes wildtype, red denotes E49D, green denotes E49A.
