# Supplementary material for: Validation of the solution structure of dimerization domain of PRC1
Source: PLoS One. 2022 Aug 5;17(8):e0270572. doi: 10.1371/journal.pone.0270572 (PMC9355583; doi:10.1371/journal.pone.0270572)
Supplement: S1 Table — (DOCX) [file pone.0270572.s012.docx]

**S1 Table.** Random-coil factor k_rc_ for every residue pair in PRC1-DD

| Residue Number | *k_rc_*(s^-1^) | Residue Number | *k_rc_*(s^-1^) |
| --- | --- | --- | --- |
| 1-2 | 215 | **34-35** | 5.2 |
| 2-3 | 13.6 | **35-36** | 13 |
| 3-4 | 26.6 | **36-37** | 2.99 |
| 4-5 | 4.23 | **37-38** | 4.84 |
| 5-6 | 0.968 | **38-39** | 13 |
| 6-7 | 1.3 | **39-40** | 9.66 |
| 7-8 | 4.22 | **40-41** | 3.36 |
| 8-9 | 2.12 | **41-42** | 0.968 |
| 9-10 | 1.5 | **42-43** | 0.989 |
| 10-11 | 11.4 | **43-44** | 4.52 |
| 11-12 | 2.54 | **44-45** | 8.22 |
| 12-13 | 0.804 | **45-46** | 10.5 |
| 13-14 | 20.7 | **46-47** | 2.35 |
| 14-15 | 6.38 | **47-48** | 3.67 |
| 15-16 | 4.84 | **48-49** | 2.79 |
| 16-17 | 9.89 | **49-50** | 1.28 |
| 17-18 | 9.02 | **50-51** | 1.11 |
| 18-19 | 1.8 | **51-52** | 2.12 |
| 19-20 | 13 | **52-53** | 4.43 |
| 20-21 | 16.6 | **53-54** | 8.61 |
| 21-22 | 3.32 | **54-55** | 1.64 |
| 22-23 | 5.07 | **55-56** | 4.03 |
| 23-24 | 3.52 | **56-57** | 2.12 |
| 24-25 | 0.904 | **57-58** | 1.5 |
| 25-26 | 1.57 | **58-59** | 1.5 |
| 26-27 | 1.64 | **59-60** | 11.4 |
| 27-28 | 1.28 | **60-61** | 3.59 |
| 28-29 | 0.786 | **61-62** | 3.85 |
| 29-30 | 7.5 | **62-63** | 2.79 |
| 30-31 | 1.88 | **63-64** | 5.83 |
| 31-32 | 1 | **64-65** | 0.0481 |
| 32-33 | 1.22 | **65-66** | 0.0481 |
| 33-34 | 2.44 |  |  |
