# Supplementary material for: Validation of the solution structure of dimerization domain of PRC1
Source: PLoS One. 2022 Aug 5;17(8):e0270572. doi: 10.1371/journal.pone.0270572 (PMC9355583; doi:10.1371/journal.pone.0270572)
Supplement: S2 Table — (DOCX) [file pone.0270572.s013.docx]

**S2 Table.** SASA values of solution and crystal structures

| Sequence | Residue | Solution Structure | Crystal Structure |
| --- | --- | --- | --- |
| 1 | MET | N/A | 50.20% |
| 2 | ARG+ | 60.80% | 52.30% |
| 3 | ARG+ | 48.50% | 67.90% |
| 4 | SER | 3.40% | 23.60% |
| 5 | GLU | 19.30% | 27.90% |
| 6 | VAL | 29.60% | 45.40% |
| 7 | LEU | 10.10% | 52.00% |
| 8 | ALA | 7.20% | 7.80% |
| 9 | GLU | 33.90% | 33.40% |
| 10 | GLU | 26.50% | 34.00% |
| 11 | SER | 0.20% | 27.60% |
| 12 | ILE | 11.40% | 32.70% |
| 13 | VAL | 36.40% | 28.70% |
| 14 | CYS | 12.70% | 18.00% |
| 15 | LEU | 0.00% | 21.10% |
| 16 | GLN | 19.60% | 39.90% |
| 17 | LYS+ | 34.50% | 42.50% |
| 18 | ALA | 5.50% | 19.00% |
| 19 | LEU | 2.80% | 31.70% |
| 20 | ASN | 17.30% | 24.80% |
| 21 | HIS+ | 11.60% | 33.70% |
| 22 | LEU | 0.40% | 25.80% |
| 23 | ARG+ | 22.00% | 29.70% |
| 24 | GLU | 24.20% | 29.50% |
| 25 | ILE | 0.00% | 16.40% |
| 26 | TRP | 2.60% | 23.20% |
| 27 | GLU | 33.30% | 37.30% |
| 28 | LEU | 37.70% | 50.10% |
| 29 | ILE | 16.70% | 32.00% |
| 30 | GLY | 45.50% | 29.90% |
| 31 | ILE | 9.60% | 22.50% |
| 32 | PRO | 28.40% | 35.20% |
| 33 | GLU | 21.70% | 32.00% |
| 34 | ASP | 28.10% | 29.70% |
| 35 | GLN | 34.20% | 23.50% |
| 36 | ARG+ | 6.30% | 9.50% |
| 37 | LEU | 6.00% | 40.90% |
| 38 | GLN | 35.30% | 35.90% |
| 39 | ARG+ | 17.30% | 30.60% |
| 40 | THR | 0.80% | 29.40% |
| 41 | GLU | 22.70% | 23.20% |
| 42 | VAL | 29.90% | 27.80% |
| 43 | VAL | 0.40% | 18.30% |
| 44 | LYS+ | 13.70% | 32.30% |
| 45 | LYS+ | 42.40% | 40.40% |
| 46 | HIS+ | 21.90% | 36.20% |
| 47 | ILE | 0.30% | 27.10% |
| 48 | LYS+ | 36.70% | 32.10% |
| 49 | GLU | 27.10% | 38.90% |
| 50 | LEU | 11.80% | 20.30% |
| 51 | LEU | 1.10% | 21.30% |
| 52 | ASP | 26.20% | 27.60% |
| 53 | MET | 38.20% | 30.50% |
| 54 | MET | 10.20% | 27.50% |
| 55 | ILE | 8.80% | 35.20% |
| 56 | ALA | 26.00% | 18.50% |
| 57 | GLU | 35.10% | 28.10% |
| 58 | GLU | 6.10% | 36.20% |
| 59 | GLU | 17.20% | 38.00% |
| 60 | SER | 19.70% | 25.30% |
| 61 | LEU | 27.60% | 30.90% |
| 62 | LYS+ | 20.00% | 33.40% |
| 63 | GLU | 42.60% | 22.00% |
| 64 | ARG+ | 48.70% | 45.50% |
| 65 | LEU | 79.10% | 52.40% |
