# Supplementary material for: Validation of the solution structure of dimerization domain of PRC1
Source: PLoS One. 2022 Aug 5;17(8):e0270572. doi: 10.1371/journal.pone.0270572 (PMC9355583; doi:10.1371/journal.pone.0270572)
Supplement: S3 Table — (DOCX) [file pone.0270572.s014.docx]

**S3 Table.** Distances of atom from residues that have variations in conformation between solution and crystal structure

| **Atom 1** | **Atom2** | **Solution Structure（Å）** | | **Crystal Structure（Å）** |
| --- | --- | --- | --- | --- |
| 1 MET HB | 110 VAL HG | 4.8 | >8 | |
| 1 MET HE | 50 LEU HD | 2.5 | >8 | |
| 1 MET HE | 50 LEU HA | 4.5 | >8 | |
| 1 MET HE | 110 VAL HG2 | 2.3 | >8 | |
| 1 MET HE | 54 MET HA | 4.9 | >8 | |
| 1 MET HE | 53 MET HB2 | 4.2 | >8 | |
| 1 MET HE | 106 ARG HB | 4.3 | >8 | |
| 18 ALA HB | 117 LEU HD1 | 2.6 | 8.5 | |
| 19 LEU HD1 | 40 THR HG | 4.9 | 6.3 | |
| 50 LEU HD1 | 53 MET HE | 3.3 | 8.6 | |
| 50 LEU HD1 | 54 MET HE | 3.4 | 6.5 | |
| 50 LEU HD1 | 51 LEU HD2 | 4.1 | 5.7 | |
| 50 LEU HD1 | 85 ALA HB | 2.5 | 8.3 | |
| 50 LEU HD2 | 82 LEU HD1 | 2.9 | 7.4 | |
| 50 LEU HD2 | 89 LEU HD1 | 2.5 | 9.3 | |
| 50 LEU HD2 | 53 MET HE | 2.5 | 6.2 | |
| 50 LEU HD2 | 54 MET HE | 4.1 | 5.8 | |
| 51 LEU HD1 | 53 MET HE | 3.8 | 9.4 | |
| 51 LEU HD1 | 54 MET HE | 2.7 | 4.9 | |
| 53 MET HE | 89 LEU HD1 | 2.4 | 9.3 | |
| 53 MET HE | 89 LEU HD2 | 2.6 | 8.5 | |
| 53 MET HE | 110 VAL HG2 | 4.9 | 11 | |
| 53 MET HE | 107 THR HN | 4.0 | 12 | |
| 54 MET HE | 55 ILE HG | 4.4 | 6.8 | |
| 54 MET HE | 55 ILE HD | 2.4 | 7.7 | |
| 54 MET HE | 85 ALA HB | 2.9 | 7.5 | |
| 57 GLU HA | 92 ILE HG2 | 3.9 | 8.4 | |
| 57 GLU HA | 96 ILE HD1 | 4.0 | 7.4 | |
| 57 GLU HB | 89 LEU HD2 | 3.0 | 6.0 | |
| 57 GLU HB | 92 ILE HG2 | 3.9 | 7.5 | |
| 57 GLU HB | 96 ILE HD1 /HG2 | 3.5 | 8.2/9.7 | |
| 57 GLU HB | 103 ARG NH1 | 3.0 | 10.5 | |
| 57 GLU HB | 106 ARG NH1 | 4.3 | 6.0 | |
