# Supplementary material for: Validation of the solution structure of dimerization domain of PRC1
Source: PLoS One. 2022 Aug 5;17(8):e0270572. doi: 10.1371/journal.pone.0270572 (PMC9355583; doi:10.1371/journal.pone.0270572)
Supplement: S4 Table — (DOCX) [file pone.0270572.s015.docx]

**S4 Table.** Table showing the primer sequences of wild-type and mutants (F is for forward primer, R is for reverse primer, restriction enzyme sites were indicated in the name of each primer)

| Construct | | | Primer | |
| --- | --- | --- | --- | --- |
| F_Nde1 | | | GGCATTCCATATGATGAGGAGAAGTGAGGTGCTG | |
| 486_R_Xhol | | | CCGCTCGAGACGTGCTTTGCCCGGTGTATT | |
| DD_R_Xhol | | | GAAAGACTCCATCATCACCATCACCATUAGCCTCGAGG | |
| R2E/F | | | AGAAGGAGATATACATATGGAGAGAAGTGAGGTGCTG | |
| R2E/R | | CAGCACCTCACTTCTCTCCATATGTATATCTCCTTCT | | |
| R2M/F | | | AGAAGGAGATATACATAGGATGAGAAGTGAGGTGCTGG | |
| R2M/R | | | CCAGCACCTCACTTCTCATCCTATGTATATCTCCTTCT | |
| N+4/F | | AGAAGGAGATATACATATGGGCGCAGCAGCAATGAGGAGAAGTGAGGTG | | |
| N+4/R | | CACCTCACTTCTCCTCATTGCTGCTGCGCCCATATGTATATCTCCTTCT | | |
| L51A/F | GCATATCAAGGAACTCGCAGATATGATGATTGCTG | | |  |
| L51A/R | CAGCAATCATCATATCTGCGAGTTCCTTGATATGC | | |  |
| L50A/F | GAAGCATATCAAGGAAGCACTGGATATGATGATTG | | |  |
| L50A/R | CAATCATCATATCCAGTGCTTCCTTGATATGCTTC | | |  |
| I55A/F | ACTCCTGGATATGATGGCAGCTGAAGAGGAAAGCC | | |  |
| I55A/R | GCTTTCCTCTTCAGCTGCCATCATATCCAGGAGT | | |  |
| E57A/F | GGATATGATGATTGCTGCAGAGGAAAGCCTGAAGG | | |  |
| E57A/R | CCTTCAGGCTTTCCTCTGCAGCAATCATCATATCC | | |  |
| E57R/F | GGATATGATGATTGCTCGAGAGGAAAGCCTGAAGG | | |  |
| E57R/R | CCTTCAGGCTTTCCTCTCGAGCAATCATCATATCC | | |  |
| E58R/F | GGATATGATGATTGCTCGAGAGGAAAGCCTGAAGG | | |  |
| E58R/R | CCTTCAGGCTTTCCTCTCGAGCAATCATCATATCC | | |  |
| E58A/F | TATGATGATTGCTGAAGCGGAAAGCCTGAAGGAAAG | | |  |
| E58A/R | CTTTCCTTCAGGCTTTCCGCTTCAGCAATCATCATA | | |  |
| E49A/F | AAAGAAGCATATCAAGGCACTCCTGGATATGATGA | | |  |
| E49A/R | TCATCATATCCAGGAGTGCCTTGATATGCTTCTTT | | |  |
