# Supplementary material for: Validation of the solution structure of dimerization domain of PRC1
Source: PLoS One. 2022 Aug 5;17(8):e0270572. doi: 10.1371/journal.pone.0270572 (PMC9355583; doi:10.1371/journal.pone.0270572)
Supplement: S5 Table — (DOCX) [file pone.0270572.s016.docx]

**S5 Table.** Table showing Residual dipolar coupling (DD) from PALES

| Resid_i | Resname | atom | Resid_j | Resname | atom | DD |
| --- | --- | --- | --- | --- | --- | --- |
| 2 | ARG | H | 2 | ARG | N | -1.8304 |
| 3 | ARG | H | 3 | ARG | N | -21.0185 |
| 4 | SER | H | 4 | SER | N | 9.4975 |
| 5 | GLU | H | 5 | GLU | N | -4.2656 |
| 7 | LEU | H | 7 | LEU | N | -38.3613 |
| 8 | ALA | H | 8 | ALA | N | -36.3407 |
| 12 | ILE | H | 12 | ILE | N | -40.4147 |
| 14 | CYS | H | 14 | CYS | N | -37.6195 |
| 15 | LEU | H | 15 | LEU | N | -37.6132 |
| 16 | GLN | H | 16 | GLN | N | -44.9802 |
| 17 | LYS | H | 17 | LYS | N | -37.5247 |
| 18 | ALA | H | 18 | ALA | N | -32.899 |
| 19 | LEU | H | 19 | LEU | N | -43.1701 |
| 20 | ASN | H | 20 | ASN | N | -44.1557 |
| 21 | HIS | H | 21 | HIS | N | -41.5679 |
| 22 | LEU | H | 22 | LEU | N | -38.9379 |
| 23 | ARG | H | 23 | ARG | N | -44.3585 |
| 24 | GLU | H | 24 | GLU | N | -42.6806 |
| 25 | ILE | H | 25 | ILE | N | -42.4122 |
| 26 | TRP | H | 26 | TRP | N | -41.3158 |
| 28 | LEU | H | 28 | LEU | N | -42.0529 |
| 30 | GLY | H | 30 | GLY | N | -22.9906 |
| 31 | ILE | H | 31 | ILE | N | -24.2947 |
| 33 | GLU | H | 33 | GLU | N | -12.0157 |
| 34 | ASP | H | 34 | ASP | N | -44.0203 |
| 35 | GLN | H | 35 | GLN | N | -24.3892 |
| 36 | ARG | H | 36 | ARG | N | -28.7594 |
| 37 | LEU | H | 37 | LEU | N | -42.0813 |
| 38 | GLN | H | 38 | GLN | N | -43.8878 |
| 44 | LYS | H | 44 | LYS | N | -35.5757 |
| 45 | LYS | H | 45 | LYS | N | -42.4314 |
| 46 | HIS | H | 46 | HIS | N | -16.9285 |
| 47 | ILE | H | 47 | ILE | N | -27.736 |
| 49 | GLU | H | 49 | GLU | N | -22.6629 |
| 50 | LEU | H | 50 | LEU | N | -23.8755 |
| 51 | LEU | H | 51 | LEU | N | -35.5034 |
| 52 | ASP | H | 52 | ASP | N | -31.0209 |
| 53 | MET | H | 53 | MET | N | -13.945 |
| 54 | MET | H | 54 | MET | N | -35.9452 |
| 56 | ALA | H | 56 | ALA | N | -24.0511 |
| 57 | GLU | H | 57 | GLU | N | -22.5553 |
| 58 | GLU | H | 58 | GLU | N | -41.9996 |
| 59 | GLU | H | 59 | GLU | N | -41.6504 |
| 60 | SER | H | 56 | SER | N | -0.3692 |
