# Supplementary material for: Validation of the solution structure of dimerization domain of PRC1
Source: PLoS One. 2022 Aug 5;17(8):e0270572. doi: 10.1371/journal.pone.0270572 (PMC9355583; doi:10.1371/journal.pone.0270572)
Supplement: S6 Table — (DOCX) [file pone.0270572.s017.docx]

**S6 Table.** Table showing RDC alignment tensors of PRC1-DD (generated by software PALES)

| Tensor | Value |
| --- | --- |
| S(zz) | 7.49E-04 |
| S(xx-yy) | -8.45E-04 |
| S(xy) | -1.64E-03 |
| S(xz) | -2.93E-04 |
| S(yz) | -2.39E-04 |
| A0 | 1.19E-03 |
| A1R | 3.80E-04 |
| A1I | -3.09E-04 |
| A2R | -5.47E-04 |
| A2I | 2.13E-03 |
| angle |  |
| ALPHA | 180.95 |
| BETA | 82.5 |
| GAMMA | 142.18 |
| Da_HN | -2.29E+01 |
| Rhombicity | 1.65E-01 |
