# Supplementary material for: Validation of the solution structure of dimerization domain of PRC1
Source: PLoS One. 2022 Aug 5;17(8):e0270572. doi: 10.1371/journal.pone.0270572 (PMC9355583; doi:10.1371/journal.pone.0270572)
Supplement: S7 Table — (DOCX) [file pone.0270572.s018.docx]

**S7 Table.** Table showing the SAXS data processed by different software, the SAXS data was collected with protein concentration of 10mg/ml for 8h, PV is for Porod Volume.

| Software | I(0) | Rg(Å) | Vc | PV | MW(Da) |
| --- | --- | --- | --- | --- | --- |
| SCATTER | 0.17 | 19.1 | 102.5 | 12151 | 14347 |
| PRIMUS | 0.18 | 20.01 | 132.7 | 19470 | 16173 |
| GNOM | 0.15 | 18.28 | 98.2 | 8608 | 17764 |
| CRYSOL | NA | 19.88 | NA | NA | 15124 |
